# Cerebellar connectome alterations and associated genetic signatures in multiple sclerosis and neuromyelitis optica spectrum disorder

**DOI:** 10.1186/s12967-023-04164-w

**Published:** 2023-05-27

**Authors:** Yuping Yang, Junle Li, Ting Li, Zhen Li, Zhizheng Zhuo, Xuemei Han, Yunyun Duan, Guanmei Cao, Fenglian Zheng, Decai Tian, Xinli Wang, Xinghu Zhang, Kuncheng Li, Fuqing Zhou, Muhua Huang, Yuxin Li, Haiqing Li, Yongmei Li, Chun Zeng, Ningnannan Zhang, Jie Sun, Chunshui Yu, Fudong Shi, Umer Asgher, Nils Muhlert, Yaou Liu, Jinhui Wang

**Affiliations:** 1 Institute for Brain Research and Rehabilitation, South China Normal University, Zhongshan Avenue West 55, Tianhe District, Guangzhou, 510631 China; 2 School of Health Sciences, Faculty of Biology, Medicine and Health, University of Manchester, Oxford Road, Manchester, M13 9PT UK; 3 Department of Radiology, Beijing Tiantan Hospital, Capital Medical University, No.119, The West Southern 4th Ring Road, Fengtai District, Beijing, 100070 China; 4 Department of Neurology, China-Japan Union Hospital of Jilin University, Changchun, 130031 Jilin China; 5 Center for Neurology, Beijing Tiantan Hospital, Capital Medical University, Beijing, 100070 China; 6 China National Clinical Research Center for Neurological Diseases, Beijing, 100070 China; 7 Department of Neurology, Tianjin Neurological Institute, Tianjin Medical University General Hospital, Tianjin, 300052 China; 8 Department of Radiology, Xuanwu Hospital, Capital Medical University, Beijing, 100053 China; 9 Department of Radiology, The First Affiliated Hospital, Nanchang University, Nanchang, 330006 Jiangxi China; 10 Neuroimaging Lab, Jiangxi Province Medical Imaging Research Institute, Nanchang, 330006 Jiangxi China; 11 Department of Radiology, Huashan Hospital, Fudan University, Shanghai, 200040 China; 12 Department of Radiology, The First Affiliated Hospital of Chongqing Medical University, Chongqing, 400016 China; 13 Department of Radiology and Tianjin Key Laboratory of Functional Imaging, Tianjin Medical University General Hospital, Tianjin, 300052 China; 14 School of Mechanical and Manufacturing Engineering (SMME), National University of Sciences and Technology (NUST), Islamabad, Pakistan; 15 Key Laboratory of Brain, Cognition and Education Sciences, Ministry of Education, Guangzhou, 510631 China; 16 Guangdong Key Laboratory of Mental Health and Cognitive Science, South China Normal University, Guangzhou, 510631 China; 17 Center for Studies of Psychological Application, South China Normal University, Guangzhou, 510631 China

**Keywords:** Multiple sclerosis, Neuromyelitis optica spectrum disorder, Cerebellum, Structural and functional MRI, Genetic correlates

## Abstract

**Background:**

The cerebellum plays key roles in the pathology of multiple sclerosis (MS) and neuromyelitis optica spectrum disorder (NMOSD), but the way in which these conditions affect how the cerebellum communicates with the rest of the brain (its connectome) and associated genetic correlates remains largely unknown.

**Methods:**

Combining multimodal MRI data from 208 MS patients, 200 NMOSD patients and 228 healthy controls and brain-wide transcriptional data, this study characterized convergent and divergent alterations in within-cerebellar and cerebello-cerebral morphological and functional connectivity in MS and NMOSD, and further explored the association between the connectivity alterations and gene expression profiles.

**Results:**

Despite numerous common alterations in the two conditions, diagnosis-specific increases in cerebellar morphological connectivity were found in MS within the cerebellar secondary motor module, and in NMOSD between cerebellar primary motor module and cerebral motor- and sensory-related areas. Both diseases also exhibited decreased functional connectivity between cerebellar motor modules and cerebral association cortices with MS-specific decreases within cerebellar secondary motor module and NMOSD-specific decreases between cerebellar motor modules and cerebral limbic and default-mode regions. Transcriptional data explained > 37.5% variance of the cerebellar functional alterations in MS with the most correlated genes enriched in signaling and ion transport-related processes and preferentially located in excitatory and inhibitory neurons. For NMOSD, similar results were found but with the most correlated genes also preferentially located in astrocytes and microglia. Finally, we showed that cerebellar connectivity can help distinguish the three groups from each other with morphological connectivity as predominant features for differentiating the patients from controls while functional connectivity for discriminating the two diseases.

**Conclusions:**

We demonstrate convergent and divergent cerebellar connectome alterations and associated transcriptomic signatures between MS and NMOSD, providing insight into shared and unique neurobiological mechanisms underlying these two diseases.

**Supplementary Information:**

The online version contains supplementary material available at 10.1186/s12967-023-04164-w.

## Background

While representing only about 10% of the size of the whole brain, the cerebellum comprises much more neurons than the cerebrum [1, 2] and contains almost 80% of the surface area of the neocortex [3]. The cerebellum has long been considered to devote exclusively to motor control, the last decade has however indicated that it is also engaged in various higher-level cognitive processes due to its complex internal structure and tight interactions with motor and non-motor regions of the cerebral cortex [4, 5]. Additionally, the cerebellum is a complex area which its connectivity and intrinsic organization interact with microscale gene expression. Recent study has suggested strong genetic correlates of within-cerebellar and cerebello-cerebral functional connectivity, with the most correlated genes involved in many neurotransmission processes and enriched in various neurological and psychiatric disorder. Furthermore, cerebellar abnormalities are continuously demonstrated to be associated with a variety of motor or non-motor dysfunctions [4, 6]. As a result, the cerebellum has attracted considerable research interests in recent years, particularly under different pathological conditions.

Multiple sclerosis (MS) and neuromyelitis optica spectrum disorder (NMOSD) are both inflammatory and demyelinating diseases which largely resulted from the interplay of environmental risk factors and genetic susceptibility. Due to their similarities in clinical presentations, correct initial diagnosis between the two diseases has remained challenging. Moreover, as the most commonly prescribed class of drug in MS, the interferon beta is ineffective and can even exacerbates clinical deterioration in NMOSD [7]. Therefore, identifying objective biomarkers that help differentiate between MS and NMOSD is of great clinical significance for timely and effective treatment. With respect to the cerebellum, widespread alterations in cerebellar structure and function have been reported in both MS [8–10] and NMOSD [11–13], but the divergence in these changes remains to be mapped out. Whilst previous studies aiming to identify cerebellar markers for differentiating between the two conditions have so far failed to find diagnosis-specific differences [14–17], these studies have exclusively focused on cerebellar alterations in local grey matter volumes, which may be less sensitive than connectivity profiles between different areas in differentiating diseases [18–21]. Understanding whether these cerebellar connectivity profiles (connectomes) are differentially altered in MS and NMOSD may shed light on condition-specific changes, and provide insight into the mechanisms underpinning these two diseases. Relatedly, there needs to be a further understanding of whether and to what extent such connectivity alterations are correlated with gene expression, which may help explore the association between macroscale connectome alterations and microscale genetic susceptibility of these two diseases.

In this study, we explored cerebellar connectome alterations and their genetic correlates in MS and NMOSD by combining multimodal MRI data collected from a large cohort of participants in seven sites and brain-wide transcriptional data from the Allen Human Brain Atlas (AHBA) dataset [22]. We hypothesized that convergent and divergent alterations would be detected in cerebellar connectome between MS and NMOSD, which were related to clinical features of patients, correlated with gene expression, and helpful for disease diagnosis and differentiation.

## Methods

### Participants

This study included a total of 752 participants including 236 patients with MS, 236 patients with NMOSD and 280 health controls (HCs) from seven sites in China. All patients were selected according to the following inclusion criteria: (1) The patients confirmed diagnosis of relapsing–remitting MS (RRMS) according to the 2017 McDonald criteria [23] or NMOSD according to 2015 revised NMOSD diagnostic criteria [24]; (2) without other neurological or psychiatric disorders; (3) right-handed; (4) complete demographic and clinical information, including age, sex, disease duration and the Expanded Disability Status Scale (EDSS) score; and (5) good quality three-dimensional (3D) T1-weighted structural images. The HCs were recruited from the local community who had no history of any clinically-diagnosed central nervous system disorders, and were aged from 18 to 65 years. Among all the participants, a total of 636 participants (208 MS patients, 200 NMOSD patients and 228 HCs) had both 3D T1-weighted structural images and resting-state functional MRI (rs-fMRI), and were finally included in this study. Additional file 1: Table S1 lists the number of participants included in each site. In the final cases, 149 MS and 147 NMOSD patients were in the remitting phase, and 48 MS and 48 NMOSD patients were in the relapsing phase. All patients received treatment in the remitting phase. Specifically, of the MS patients, 86 (41.3%) received an MS-specific disease-modifying therapy (DMT), and the others received immunosuppressants including cyclophosphamide and azathioprine. Regarding the NMOSD patients, 52 (26.0%) received DMT, and the others received the above other treatment. Of the NMOSD patients, 139 (69.5%) were tested for AQP4-Ab (AQP4-Ab positive: 90; AQP4-Ab negative: 49), and 25 (12.5%) were tested for MOG antibody using the cell-based assay [25]. This study was approved by the institutional review board of corresponding hospitals, and written informed consent was obtained from each participant.

### Clinical and neuropsychological assessment

Clinical variables included disease duration and EDSS score. Neuropsychological assessment included the California Verbal Learning Test-Second Edition (CVLT), Brief Visuospatial Memory Test-Revised (BVMT) and Paced Auditory Serial Addition Task-3 seconds (PASAT). Of note, only a small subset of participants in three sites completed certain neuropsychological tests, which were performed when the patients were in either the relapsing or remitting phase of the diseases (Additional file 1: Table S2).

### Multimodal MRI data acquisition

Multimodal images were acquired on 3.0 T scanners, including T2-weighted FLAIR imaging, high-resolution 3D T1-weighted imaging and rs-fMRI. For the patients in the relapsing phase, the MRI was performed after acute treatment. The imaging parameters are summarized in Additional file 1: Table S3.

### White matter lesion loads and structural image filling

For each patient, a white matter (WM) lesion mask was manually delineated using the 3D-slicer software (https://www.slicer.org) if WM hyperintensity was evident on T2-weighted FLAIR images. Individual WM lesion loads were calculated as the total volumes within the masks. Based on the masks, the superior longitudinal fasciculus (SLF) algorithm was used to refill WM lesions on individual T1-weighted images (http://atc.udg.edu/salem/slfToolbox/software.html).

### Structural and functional images processing

The structural images preprocessing and cortical thickness estimation of the cerebellum and cerebrum were accomplished with the CERES toolbox [26] on the volBrain online pipeline [27] and CAT12 toolbox (http://www.neuro.uni-jena.de/cat/), respectively. The functional images preprocessing was performed with the SPM12 toolbox (https://www.fil.ion.ucl.ac.uk/spm/software/spm12/). See Additional file 1 for details.

### Construction of region-level within-cerebellar and cerebello-cerebral networks

#### Region-level parcellation

The cerebellum was segmented into 24 regions of interest (ROIs) using a multi-atlas segmentation CERES toolbox [26] (Additional file 1: Fig S1). The cerebrum was divided into 400 ROIs through the functionally-defined Schaefer atlas [28].

#### Region-level connectivity estimation

For a given pair of regions within the cerebellum or between the cerebellum and cerebrum, morphological connectivity was estimated by calculating Jensen-Shannon divergence-based similarity in regional cortical thickness distribution [29, 30] using in-house codes based on GRETNA toolbox [31]; functional connectivity was estimated by calculating Pearson correlation in regional mean time series. For the resulting connectivity matrices, Combat harmonization [32] was used to moderate the site effects. See Additional file 1 for details.

### Cerebellar module detection

In this study, cerebellar module architecture was identified by applying a multilayer module detection algorithm to a multiplex network [33], which integrated morphological and functional connectivity within the cerebellum for the HCs. Two parameters may influence the outcome during the community detection: the inter-layer connectivity strength, $\omega$, and the module resolution, $\gamma$. To identify stable cerebellar modular architecture, we performed community detection across a wide range in the 2D parameter space of ($\omega$,$\gamma$): $\omega$ = [0.01–1] and $\gamma$ = [0.01–3], and employed the variation of information [34] to evaluate the stability of the resultant module architecture over different ($\omega$,$\gamma$) (Additional file 1: Fig S2). See Additional file 1 for details.

### Cerebral module definition

The cerebrum was divided into canonical modules based on two approaches: (1) Cytoarchitectonic classification. The cerebral cortex displays substantial variation in cytoarchitecture, according to which the cerebrum is categorized into seven cytoarchitectonic modules [35, 36]: primary motor cortex (PM), association cortex (AC1), association cortex (AC2), primary/secondary sensory (PSS), primary sensory cortex (PS), limbic regions (LB) and insular cortex (IC); 2) Functional subnetwork. In terms of intrinsic functional connectivity patterns, the cerebrum was divided into seven functional modules [37]: visual network (VN), somatomotor network (SMN), dorsal attention network (DAN), ventral attention network (VAN), limbic network (LN), frontoparietal network (FPN) and default mode network (DMN). Each of the 400 cerebral ROIs was assigned to one of the seven cytoarchitectonic/functional modules according to the maximum overlap in terms of voxel amounts.

### Conversion of cerebellar networks from region-level to module-level

Based on the detected cerebellar modules and the prior defined cerebral cytoarchitectonic/functional modules, the region-level cerebellar networks derived above were transformed into module-level networks, including module-level within-cerebellar networks and module-level cerebello-cerebral networks. See Additional file 1 for details.

### Statistical analysis

#### Between-group differences

Chi-squared tests were used to compare dichotomous variables. Non-parametric permutation tests (10,000 times) were used to compare continuous variables due to their non-normal distributions (Lilliefors test). Age, sex and mean framewise displacement of head motion (if applicable) were treated as covariates for comparisons of neuropsychological variables and imaging-based measurements. Multiple comparisons were corrected with the false discovery rate (FDR) procedure. For significant differences among the three groups, post hoc pairwise comparisons were further performed by non-parametric permutation tests (10,000 times). See Additional file 1 for details.

#### Relationship between cerebellar image-based measures and other variables

Spearman partial correlation was used to examine the relationships of cerebellar connectivity alterations with clinical data, neuropsychological tests and lesion volumes in the MS and/or NMOSD patients with age, sex and mean framewise displacement of head motion (if applicable) as covariates. Multiple comparisons were corrected by the FDR procedure.

### Effects of disease phase

In this study, both the relapsing and remitting patients were included. To examine potential effects of disease phase on the between-group differences, we further compared the neuropsychological variables and imaging-based measurements showing disease-related alterations between the relapsing and remitting patients for each disease (non-parametric permutation tests; 10,000 times). Age, sex and mean framewise displacement of head motion (if applicable) were treated as covariates. Multiple comparisons were corrected with the FDR approach.

### Genetic correlates of cerebellar connectivity alterations in MS and NMOSD

We used gene data from the AHBA to explore genetic correlates of cerebellar connectivity alterations in MS and NMOSD. The AHBA provides transcriptional activity of 20,737 genes from 3702 spatially distinct tissue samples that were collected from six healthy adult human donors. After standardized processing workflows [38], we obtained group-level gene expression profiles for each ROI, quantifying the mean transcriptional activity of 15,631 genes, which were used to calculate gene co-expression (Pearson correlation) between each pair of regions within the cerebellum and for each pair of regions between the cerebellum and cerebrum. The gene co-expression networks further underwent region-level-to-module-level transformation. To explore genetic correlates of observed cerebellar connectivity alterations, we evaluated the contributions of each gene to the gene co-expression networks, which were used as predictor variables to explain the variations in cerebellar connectivity alterations in the patients via partial least-squares (PLS) regression. The first component of the PLS (PLS1) was the linear combination of the contributions of all genes to the gene co-expression networks that exhibited the strongest correlation with cerebellar connectivity alterations. The weight of each gene to form the PLS1 was converted to a Z score by subtracting the mean value and dividing by the standard deviation of all gene weights, and genes with an absolute Z score greater than 1.64 were considered to significantly related to cerebellar connectivity alterations. To better understand the identified genes, we further performed gene ontology (GO) functional enrichment analysis using the online tool GOrilla (http://cbl-gorilla.cs.technion.ac.il, version 27 Mar 2022) [39] to identify the gene-related biological processes, and examined the cell-type specificity in the spatial expression distribution of the identified genes in seven canonical cell classes [40]. See Additional file 1 for details.

### Classification analysis

We trained linear SVM classifiers to distinguish the three groups from each other based on cerebellar connectivity profiles. Out-of-sample classification performance was evaluated using a tenfold cross-validation procedure. The procedure was repeated 100 times and the accuracy values were averaged for robust estimation of classification performance, which was further compared with random operations (1000 times) to obtain the significance level. See Additional file 1 for details.

## Results

### Demographic, clinical, and neuropsychological evaluation

Table 1 summarizes demographic, clinical and neuropsychological information of the participants. Significant group effects were found for age (*p* = 0.003) and sex (*p* < 0.001). The NMOSD patients exhibited older ages than both MS patients (*p* < 0.001) and HCs (*p* = 0.020), and had higher female-to-male ratios than both MS patients (*p* < 0.001) and HCs (*p* < 0.001); there was also a higher female-to-male ratio for MS patients compared to HCs (*p* = 0.025). For clinical data, no significant differences were found in the relapsing-to-remitting phase ratio or disease duration between the two patient groups (*p* > 0.05). However, the NMOSD patients exhibited fewer WM lesions (*p* < 0.001) and lower total lesion volumes (*p* < 0.001) than the MS patients. In addition, the NMOSD patients showed significantly higher EDSS than the MS patients (*p* < 0.001). Regarding neuropsychological variables, both the patient groups performed significantly worse than the HCs in the CVLT and PASAT (both *p* < 0.001). In addition, the NMOSD patients showed lower BVMT than the HCs (*p* < 0.001). No differences were found in any neuropsychological variable between the two patient groups (*p* > 0.05).

**Table 1:** Demographic, clinical and neuropsychological variables

	HCs (n = 228)	MS (n = 208)	NMOSD (n = 200)	*p*-value
Age (years)	37.0 (21.0)	36.0 (17.0)	41.0 (21.5)	0.003^a,c^
Sex (female/male)	124/104	135/73	175/25	< 0.001^a,b,c^
Disease state (relapsing/remitting)^*^	–	48/149	48/147	0.954
Disease duration (months)	–	19.0 (54.0)	36.0 (52.0)	0.054
Lesion				
N (%)	–	208 (100%)	110 (55%)	< 0.001
Volume (cm^3^)	–	7.6 (16.0)	1.2 (4.0)	< 0.001
EDSS	–	2.0 (2.5)	3.5 (3.0)	< 0.001
CVLT^†^	52.0 (10.8)	47.5 (15.0)	47.0 (14.5)	< 0.001^b,c^
PASAT^†^	51.5 (14.0)	40.0 (17.0)	40.0 (18.0)	< 0.001^b,c^
BVMT^†^	28.0 (5.8)	27.0 (11.8)	22.0 (12.0)	0.003^c^

Data are represented as median (interquartile range) unless stated otherwise

*HCs* healthy controls, *MS* multiple sclerosis, *NMOSD* neuromyelitis optica spectrum disorders, *EDSS* Expanded Disability Status Scale, *CVLT* California Verbal Learning Test, *PASAT* Paced Auditory Serial Addition Test, *BVMT* Brief Visuospatial Memory Test

^*^Data are missing for 16 patients

^†^Data are available only for a subset of participants from three hospitals (see Materials and Methods for details)

^a^Significant differences between the two patient groups

^b^Significant differences between the MS patients and HCs

^c^Significant differences between the NMOSD patients and HCs

### Cerebellar modular architecture

**Fig. 1:**
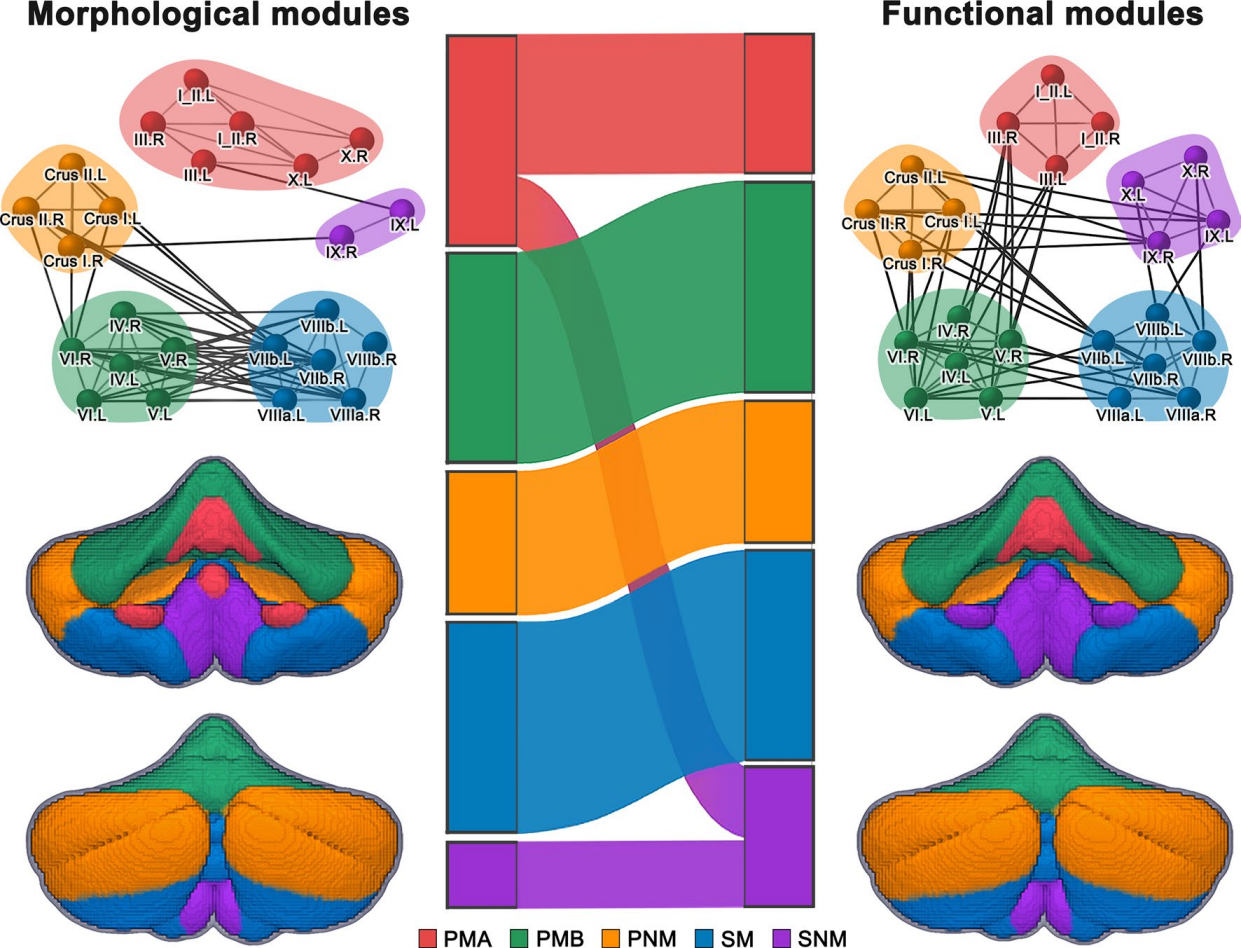
Modular architecture of the cerebellum. Five cerebellar modules were identified by applying a multilayer community detection algorithm to the group-level multiplex network of the HCs that integrated morphological and functional connectivity within the cerebellum. Module assignments of cerebellar lobules were largely comparable between morphological and functional networks. *PMA* Primary Motor A, *PMB* Primary Motor B, *PNM* Primary Non-Motor, *SM* Secondary Motor, *SNM* Secondary Non-Motor

The cerebellum was subdivided into five spatially contiguous and bilaterally symmetrical modules (*Q* = 0.566; Fig. 1). The modules had a good correspondence with the well-established cerebellar double-motor/triple-non-motor organization, and therefore were termed Primary Motor A module (PMA, including bilateral Lobule I-II and Lobule III, with additional bilateral Lobule X for morphological networks), Primary Motor B module (PMB, including bilateral Lobule IV, Lobule V and Lobule VI), Primary Non-Motor module (PNM, including bilateral Crus I and Crus II), Secondary Motor module (SM, including bilateral Lobule VIIb, Lobule VIIIa and Lobule VIIIb) and Secondary Non-Motor module (SNM, including bilateral lobule IX, with additional bilateral Lobule X for functional networks). See Additional file 1 for detailed results and relevant discussion.

### Alterations in cerebellar morphological connectivity

**Fig. 2:**
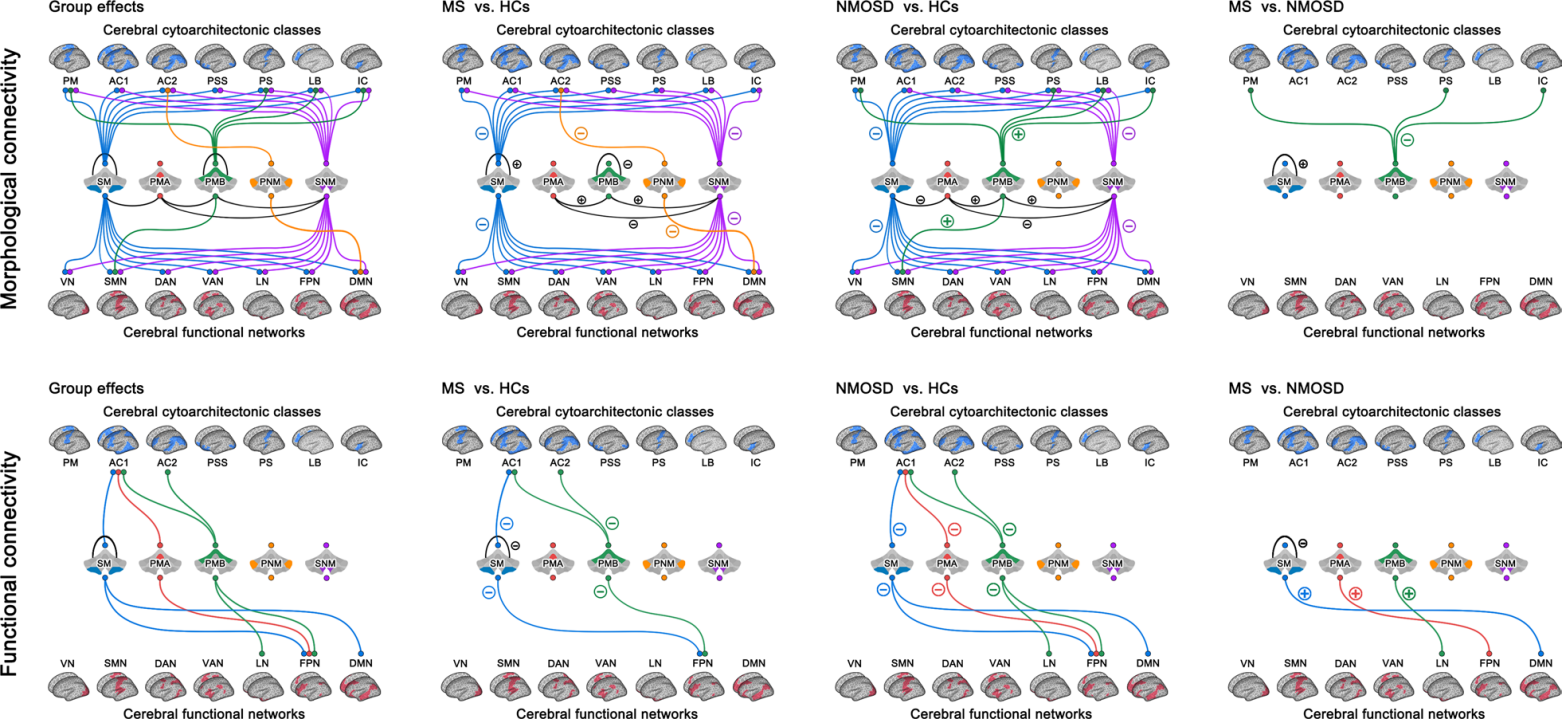
Alterations in cerebellar module-based morphological and functional connectivity. For morphological connectivity, numerous alterations of both increases and decreases were found in the two patient groups. Compared with morphological connectivity, fewer alterations were observed for functional connectivity in the two patient groups that were all characterized by disease-related decreases in particular for connectivity between cerebellar motor modules and cerebral association cortex or high-order networks. *HCs* healthy controls, *MS* multiple sclerosis, *NMOSD* neuromyelitis optica spectrum disorders, *PMA* Primary Motor A, *PMB* Primary Motor B, *PNM* Primary Non-Motor, *SM* Secondary Motor, *SNM* Secondary Non-Motor, *PM* primary motor cortex, *AC1* association cortex, *AC2* association cortex, *PSS* primary/secondary sensory, *PS* primary sensory cortex, *LB* limbic regions, *IC* insular cortex, *VN* visual network, *SMN* somatomotor network, *DAN* dorsal attention network, *VAN* ventral attention network, *LN* limbic network, *FPN* frontoparietal network, *DMN* default mode network

Differences in cerebellar morphological connectivity are shown in Fig. 2 (top panel) and Additional file 1: Table S4.

#### Within-cerebellar morphological connectivity

Significant group effects were mainly found in mean morphological connectivity within the cerebellar SM and between three pairs of cerebellar modules (PMA—SNM, PMA—PMB and SNM—PMB) (*p* < 0.05, FDR corrected). These group effects were mainly owing to MS-specific increases (MS > [NMOSD = HCs]: SM) as well as common increases ([MS = NMOSD] > HCs: PMA—PMB and SNM—PMB) and common decreases ([MS = NMOSD] < HCs: PMA—SNM) in the two patient groups.

#### Cerebello-cerebral morphological connectivity

Significant group effects were found in mean morphological connectivity between cerebellar modules and cerebral cytoarchitectonic modules, including the connectivity for the cerebellar SM and SNM with all cerebral cytoarchitectonic classes, cerebellar PMB with cerebral PM, PS, LB and IC, and cerebellar PNM with cerebral AC2 (*p* < 0.05, FDR corrected). Post hoc comparisons revealed that these group effects were owing to common decreases in the two patient groups ([MS = NMOSD] < HCs: SM—all cerebral cytoarchitectonic classes, and SNM—AC1, AC2, PSS, PS and LB), NMOSD-specific increases (NMOSD > [MS = HCs]: PMB—PM, PS and IC), NMOSD-related increases (NMOSD > HCs: PMB—LB), or MS-related decreases (MS < HCs: PNM—AC2, and SNM—PM and IC). Significant group effects were also found in mean morphological connectivity between cerebellar modules and cerebral functional modules, including the connectivity for the cerebellar SM and SNM with all cerebral functional systems, cerebellar PMB with cerebral SMN, and cerebellar PNM with cerebral DMN (*p* < 0.05, FDR corrected). These group effects were owing to common decreases in the two patient groups ([MS = NMOSD] < HCs: SM and SNM—all cerebral functional systems), NMOSD-related increases (NMOSD > HCs: PMB—SMN), or MS-related decreases (MS < HCs: PNM—DMN).

### Alterations in cerebellar functional connectivity

Differences in cerebellar functional connectivity are summarized in Fig. 2 (bottom panel) and Additional file 1: Table S4.

#### Within-cerebellar functional connectivity

Significant group effects were found in the functional connectivity only within the cerebellar SM (*p* < 0.05, FDR corrected) due to MS-specific decreases (MS < [NMOSD = HCs]).

#### Cerebello-cerebral functional connectivity

Significant group effects were found in mean functional connectivity between cerebellar modules and cerebral cytoarchitectonic modules, including the connectivity for the cerebellar PMB with cerebral AC1 and AC2, cerebellar SM with cerebral AC1, and cerebellar PMA with cerebral AC1 (*p* < 0.05, FDR corrected). Post hoc comparisons revealed that the group effects were owing to common decreases in the two patient groups ([MS = NMOSD] < HCs: PMB—AC1 and AC2, and SM—AC1), or NMOSD-related decreases (NMOSD < HCs: PMA—AC1). Significant group effects were also found in mean functional connectivity between cerebellar modules and cerebral functional modules, including the connectivity for the cerebellar PMB with cerebral FPN and LN, cerebellar SM with cerebral FPN and DMN, and cerebellar PMA with cerebral FPN (*p* < 0.05, FDR corrected). These group effects were owing to common decreases in the two patient groups ([MS = NMOSD] < HCs: PMB—FPN, and SM—FPN), or NMOSD-specific decreases (NMOSD < [MS = HCs]: PMA—FPN, PMB—LN, and SM—DMN).

### Differences in neuropsychological tests and cerebellar connectivity between the relapsing and remitting phase

For the neuropsychological variables showing disease-related alterations (MS: 2; NMOSD: 3), significant effects of disease phase were observed only on the PASAT in NMOSD with lower scores in the remitting than relapsing patients (*p* = 0.003). With regard to cerebellar morphological (MS: 35; NMOSD: 35) and functional (MS: 6; NMOSD: 9) connectivity showing disease-related alterations, significant effects of disease phase were observed only on the morphological connectivity within the cerebellar PMB (relapsing > remitting) in MS, and on the functional connectivity between the cerebellar PMB and cerebral LN (relapsing < remitting) in NMOSD (*p* < 0.05, FDR corrected). Notably, when we further expanded the comparisons to all cerebellar connectivity instead of only focusing on those showing disease-related alterations, no additional effects of disease phase were observed. These findings suggest that the cerebellar connectivity are largely comparable between relapsing and remitting patients, and thus the observed disease-related alterations in cerebellar connectivity seem to be not caused by an acute attack but rather more likely reflect the long-term cumulative impact of the diseases.

### Relationship between abnormal cerebellar connectivity and clinical/neuropsychological variables

No significant correlations were observed for cerebellar module-based connectivity alterations with any clinical or neuropsychological variables or lesion volume in the two patient groups (*p* > 0.05, FDR corrected).

### Genetic substrates of cerebellar functional connectivity alterations in MS and NMOSD

**Fig. 3:**
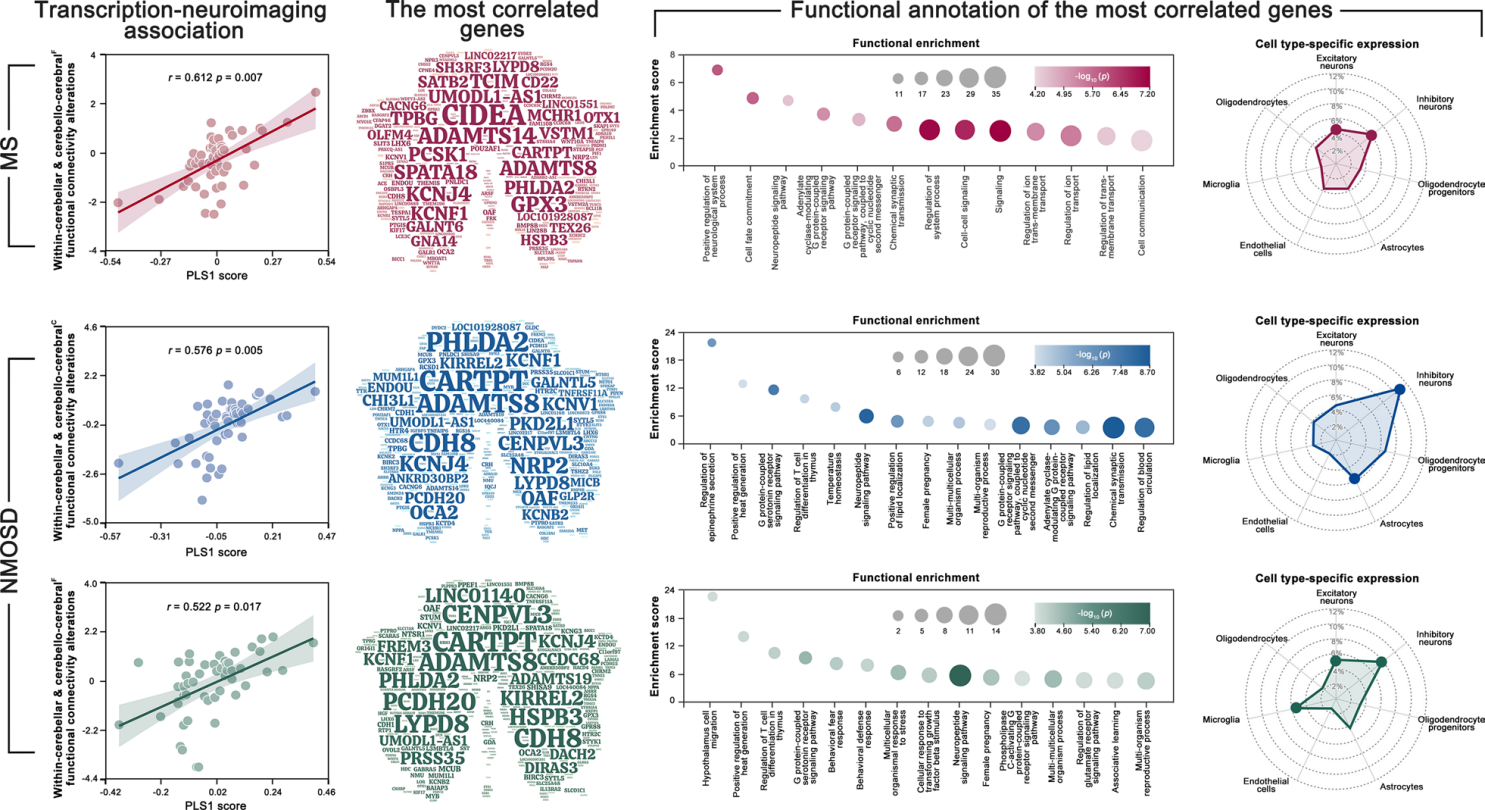
Genetic correlates of cerebellar functional connectivity alterations. Transcriptional data explained > 37.5% variance of the cerebellar functional connectivity alterations in MS with the most correlated genes enriched in signaling and ion transport-related processes and preferentially located in excitatory and inhibitory neurons. For NMOSD, around 30% variance of the cerebellar functional connectivity alterations were explained by transcriptional profiles with the most correlated genes enriched in female pregnancy and multi-organism reproductive process in addition to signaling and ion transport-related processes and preferentially located in astrocytes and microglia in addition to excitatory and inhibitory neurons. MS, multiple sclerosis; NMOSD, neuromyelitis optica spectrum disorders. *Cerebral*^*C*^ cerebral cytoarchitectonic modules, *Cerebral*^*F*^ cerebral functional modules

Cerebellar genetic substrates exhibited a significantly positive correlation with functional connectivity alterations within the cerebellum and between the cerebellar modules and cerebral functional modules in the MS patients (*r* = 0.612, *p* = 0.007). The most relevant genes were mainly related to signaling and ion transport-related biological processes and were preferentially located in specific cell types of excitatory and inhibitory neurons (*p* < 0.05, FDR corrected) (Fig. 3 top panel, Additional file 1: Table S5). For NMOSD, cerebellar genetic substrates also exhibited a significantly positive correlation with functional connectivity alterations within the cerebellum and between the cerebellar modules and cerebral modules (cytoarchitectonic/functional modules: *r* = 0.576/0.522, *p* = 0.005/0.017). The most relevant genes were associated with female pregnancy and multi-organism reproductive process in addition to signaling and ion transport-related biological processes, and were preferentially located in specific cell types of astrocytes and microglia besides excitatory and inhibitory neurons (*p* < 0.05, FDR corrected) (Fig. 3 middle/bottom panel, Additional file 1: Table S5).

### Group classification

**Fig. 4:**
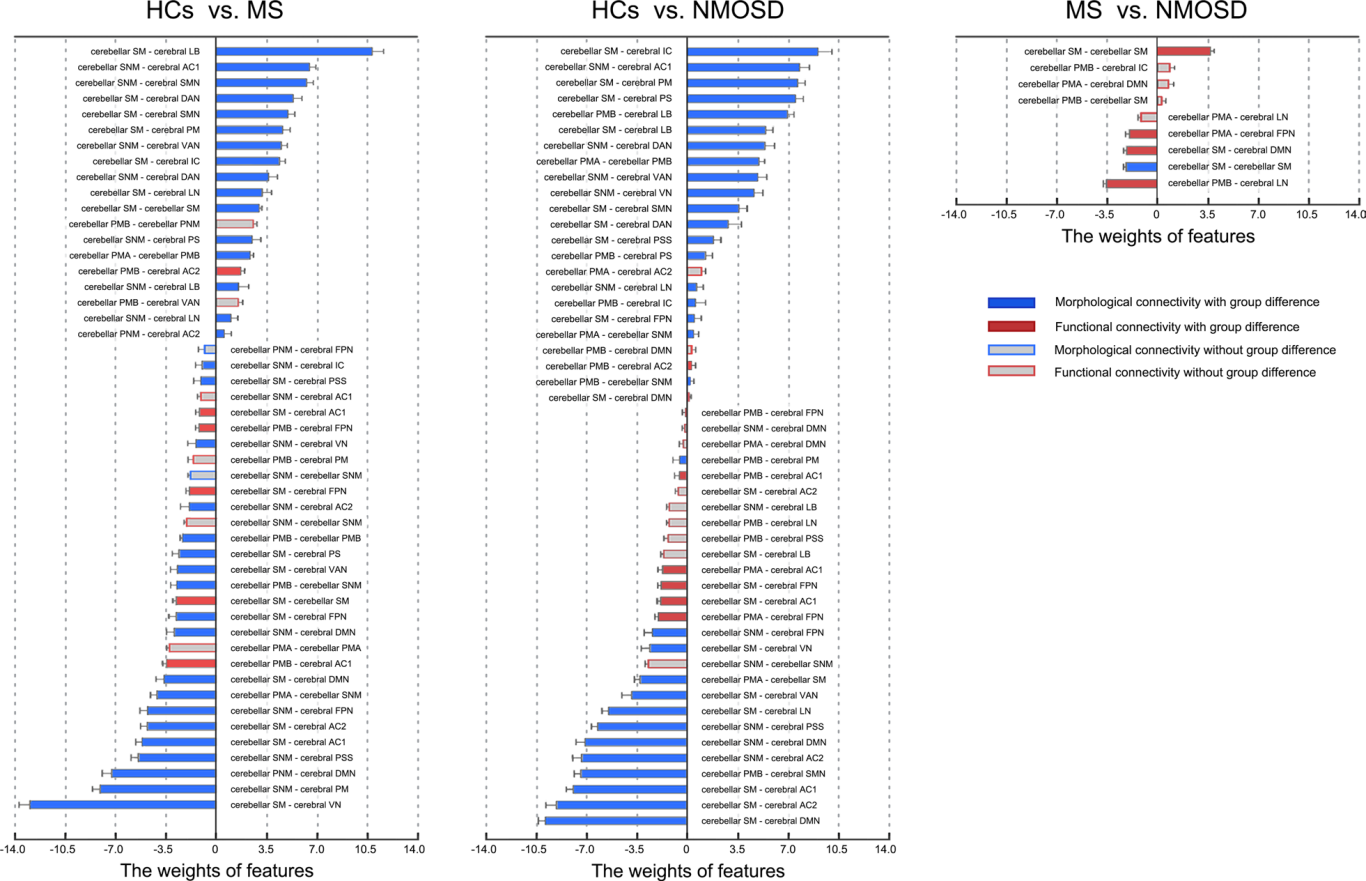
Features contributing to the classification between groups. The features contributing to the classification were mainly composed of connectivity that exhibited significant between-group differences. The classification between the patients and HCs mainly relied on morphological connectivity in particular those involving the PMB, SM and SNM, while the classification between the two diseases mainly benefited from functional connectivity that were all involved in the three motor-related modules (i.e., the PMA, PMB and SM). *HCs* healthy controls, *MS* multiple sclerosis, *NMOSD* neuromyelitis optica spectrum disorders, *PMA* Primary Motor A, *PMB* Primary Motor B, *PNM* Primary Non-Motor, *SM* Secondary Motor, *SNM* Secondary Non-Motor, *PM* primary motor cortex, *AC1* association cortex, *AC2* association cortex, *PSS* primary/secondary sensory, *PS* primary sensory cortex, *LB* limbic regions, *IC* insular cortex, *VN* visual network, *SMN* somatomotor network, *DAN* dorsal attention network, *VAN* ventral attention network, *LN* limbic network, *FPN* frontoparietal network, *DMN* default mode network

The cerebellar connectivity distinguished the three groups from each other with around 60% accuracies (MS vs HCs: 63.3%, *p* < 0.001; NMOSD vs HCs: 64.4%, *p* < 0.001; MS vs NMOSD: 57.9%, *p* = 0.001). As shown in Fig. 4, morphological connectivity predominated in features contributing to the differentiation between the patients and HCs (MS vs HCs: 75.5%; NMOSD vs HCs: 64.0%) while the classification between the two diseases mainly benefited from functional connectivity (MS vs NMOSD: 88.9%). In the context of cerebellar modular architecture, the morphological connectivity contributing to the classification between the patients and HCs were mainly related to the PMB, SM and SNM (MS vs HCs: 91.9%; NMOSD vs HCs: 100%). For classifying the two patient groups, the functional connectivity contributing to the classification were all involved in the three motor-related modules (i.e., the PMA, PMB and SM; MS vs NMOSD: 100%).

### Alterations in cerebellar cortical thickness

In addition to the cerebellar connectivity, we examined cerebellar cortical thickness, which exhibited disease-related alterations and clinical associations (Additional file 1: Fig S3, S4). See Additional file 1 for detailed results and discussion.

## Discussion

In this study, we investigated cerebellar connectome alterations and their genetic correlates in MS and NMOSD. Convergent and divergent alterations in within-cerebellar and cerebello-cerebral morphological and functional connectivity were detected in MS and NMOSD, among which the pathological alterations in functional, but not morphological, connectivity were associated with cell type-specific gene expression. These findings indicate network-level cerebellar dysfunctions in MS and NMOSD, and provide insights into molecular mechanisms underlying the cerebellar dysfunctions of the two diseases.

### Alterations in cerebellar morphological connectivity

We found that the patients with MS and NMOSD exhibited common decreases for the SM and SNM with almost all the cerebral components. The cerebellum is closely linked with the cerebrum via complex cerebello-cerebral loops to exchange information with each other and participate collectively in various motor and non-motor activities. The widely disrupted cerebello-cerebral morphological loops may relate to poor performance on a set of motor and non-motor domains in MS and NMOSD. Notably, the disruptions seem to be specific to the secondary rather than primary modules. Previous studies have suggested that the secondary modules, compared with the primary modules, are engaged in motor processes that require higher attention, working memory and sometimes visual process rather than pure movements, and are devoted to those non-motor processes involving multiple cognitive and affective activities rather than single non-motor domain [4, 41]. Taking these facts into consideration, we could reasonably deduce that the numerous disruptions between cerebellar secondary modules and the cerebrum might account for the well described deficits in those compounded motor and non-motor activities in MS and NMOSD [42, 43]. In addition to the common decreases, we found morphological connectivity reductions for the cerebellar PNM with the cerebral AC2 and DMN in the MS patients. The cerebellar PNM is constituted of the bilateral Crus I and Crus II, which are key cognitive lobules in the cerebellum. Previous studies have indicated that both the Crus I and Crus II have close connections with cerebral association cortex [44, 45], and lesions affecting the Crus I and Crus II lead to cognitive impairments by interrupting cerebellar regulation of cognitive loops with cerebral association cortex [5]. On the other hand, the Crus I and Crus II are found to be major sites to interact with the cerebral DMN [46], and damages to cerebello-cerebral DMN connections are related to cognitive impairments in MS patients [47]. Accordingly, we speculate that the disrupted morphological connectivity between the cerebellar PNM and cerebral AC2 and DMN may contribute to a variety of cognitive deficits in MS patients. Finally, we found NMOSD-specific enhancement between the cerebellar PMB module and cerebral PM, PS, IC and SMN. Given that the components in both the cerebellar PMB module and cerebral PM, PS, IC and SMN play important roles in motor and/or sensory processing, this enhancement might therefore reflect compensatory response to NMOSD-specific cortical damage in the PMB module to indemnify motor and sensory functions in patients.

### Alterations in cerebellar functional connectivity

Compared with numerous morphological connectivity alterations, much less alterations were detected in functional connectivity in the patients. Moreover, the functional connectivity alterations were all characterized by decreases in the patients and mainly involved cerebellar motor modules and cerebral association cortices. Specifically, MS-specific decreases were observed in the mean functional connectivity of cerebellar SM module. According to the functional network collapse theory in MS [48, 49], these module-level functional connectivity changes may reflect a functional maladaptation caused by the widespread damage on morphological coherences not only within the cerebellar SM module but also between this module and all the cerebral modules. Notably, given the relatively short disease duration of the MS patients in this study, the usage of network collapse theory to explain the association between functional network changes and structural/morphological damage can only be speculative. With regard to cerebello-cerebral functional connectivity, both the patient groups showed decreases for the cerebellar PMB and SM with the cerebral association cortex and FPN. The cerebellum is closely related with both the cerebral association cortex and the FPN. On the one hand, the newest parts of the cerebellum (e.g., the posterior lobe that includes the SM) develops specifically in parallel with cerebral association cortex rather than the cerebral cortex as a whole [50], facilitating the coordination between the cerebellum and cerebral association areas to exchange highly-processed multisensory information and cooperate in high-level functions [51]. On the other hand, the FPN, which is overrepresented in the cerebellum [52] with components overlapping the PMB and SM, modulates the integration of association and motor networks [53]. Together, the disrupted functional connectivity suggests failed or weakened information exchange between the cerebellar motor modules and the cerebral association cortex as well as FPN in supporting motor, multisensory and high-level processes in MS and NMOSD.

### Genetic substrates underlying cerebellar functional connectivity alterations in MS and NMOSD

Significantly positive genetic correlations were found for cerebellar functional but not morphological connectivity alterations in the two diseases, suggesting genetic substrates of integrative dysfunctions of cerebellar functional connectivity in MS and NMOSD. The relevant genes in the two diseases were mainly enriched in signaling and ion transport-related biological processes, which play crucial roles in transmitting biological information and maintaining cell homeostasis. Previous studies have shown that MS and NMOSD are related to disrupted ion channels [54, 55]. Moreover, signaling and ion transport-related dysfunctions can lead to a variety of clinical symptoms that are frequently observed in MS and NMOSD including memory loss, neuromuscular sprains and motor-related disabilities [56]. Accordingly, the genes enriched in the signaling and ion transport-related biological processes may, at least partly, account for common cerebellar functional connectivity alterations and clinical manifestations to MS and NMOSD. In addition, our functional annotation analysis revealed several Go terms specific to NMOSD, such as female pregnancy and multi-organism reproductive processes. These biological processes run through the period of the embryonic development and contribute to producing new individuals. In NMOSD, the prevalence shows a strong female bias, especially among patients aged 15–40 years, a typical period of pregnancy and reproduction [57]. Previous studies have shown that NMOSD patients were associated with an increased relapse rate during the period of pregnancy and postpartum, and exhibited a higher risk of miscarriage relative to healthy individuals [58, 59]. We therefore speculate that the genes enriched in the NMOSD-specific biological processes are responsible for unique cerebellar functional connectivity alterations and characteristics of the disease.

Further, we found that the relevant genes in MS and NMOSD showed predilections in terms of cellular architecture. Specifically, the genes were preferentially located in the excitatory and inhibitory neurons in both MS and NMOSD. These two types of neurons are responsible for the transmission and regulation of nerve impulses. Previous studies have reported imbalance between excitatory and inhibitory transmission in both MS [60] and NMOSD [61]. Therefore, the transdiagnostic preferences to excitatory and inhibitory neurons may imply the key roles of abnormal nerve impulses in integrative dysfunctions of cerebellar functional connectivity in MS and NMOSD. Besides the transdiagnostic predilections, the relevant genes in NMOSD showed additional preference to astrocytes and microglia. As the most common target antigen in NMOSD, the AQP4 is concentrated in the central nervous system at astrocyte terminal protrusions. In a recent mouse study, it was found that AQP4-IgG induced direct interaction between microglia and astrocytes, which may be a critical driver of the evolving NMO lesion and the emergence of motor impairments [62]. These findings suggest important implications of astrocyte-microglia crosstalk for the pathogenesis of NMOSD. In the future, a better understanding of the roles of astrocytes and microglia in NMOSD may benefit from studies that directly associate the microscopic processes derived from animal models with neuroimaging-based features derived from human patients with the help of advanced computational modeling and cross-species mapping.

### The potential of cerebellar connectivity as biomarkers for classification

Our classification results indicated that cerebellar morphological connectivity had the potential to help distinguish the patients from controls while functional connectivity for distinguishing the two diseases from each other. The discrepancy is consistent with previous findings that different types of connectivity have poor correspondences [63]. Notably, the classification accuracies were relatively low. Clinical heterogeneity of the patients, a critical concern for retrospective large-scale multisite studies, may be a main reason for the low accuracies. In addition, more sophisticated deep learning algorithms, such as convolutional neural network, may further improve the accuracies. Finally, we noted that certain cerebellar modules dominated the connectivity features in the classification. Future studies can improve the classification by exclusively focusing on a specific set of regions or connections that are closely related to the diseases.

## Limitations

This study has several limitations. First, all patients included in this study received treatment. A previous study found that intramuscular interferon beta-1a treatment can significantly slow the progression of brain atrophy in relapsing–remitting MS [64], indicative of treatment effects on brain volume. However, the methods and degree of treatment differed largely between patients in this retrospective multicentric study. This prevents us from excluding or weakening the treatment effects on our findings by image post-processing techniques or statistical models. Future prospective studies are needed to explore whether and to what extent different treatment methods affect cerebellar connectome by using longitudinal design. Second, only a small subset of participants in three sites completed certain neuropsychological tests. Therefore, all results involving the neuropsychological data should be explained with cautions since the data may be insufficient to reflect the cognitive characteristics of the entire population. Future large-sample studies with complete and uniform neuropsychological tests are needed to examine whether cognitive abilities of MS and NMOSD patients differ between different phases of the diseases, and how disturbances in the abilities are related to cerebellar connectivity alterations. Third, although we used the Combat harmonization to mitigate site effects for the retrospective design of this multisite study, it is still not clear regarding the extent to which our findings are affected by residual site effects. Finally, MS and NMOSD are heterogeneous diseases, both of which can be divided into different phenotypes based on clinical evolution. Future studies are needed to explore subtype-specific cerebellar network alterations in MS and NMOSD.

## Conclusion

This study provided the evidence of convergent and divergent alterations in within-cerebellar and cerebello-cerebral connectivity as well as their associated transcriptomic signatures in MS and NMOSD, suggesting novel insights into shared and unique neurobiological mechanisms underlying the two diseases.

## Supplementary Information

**Additional file 1: Supplementary Methods. Supplementary Results. Supplementary Discussion.** **Figure S1.** Illustrative representation of cerebellar lobules. **Figure S2.** The stability of cerebellar modular architecture over different combinations of inter-layer connectivity strength ($\upomega$) and module resolution ($\upgamma$). **Figure S3.** Alterations in cerebellar module-based cortical thickness. **Figure S4.** Relationships between cerebellar module-based cortical thickness and disease duration, lesion volume and EDSS in MS. **Table S1.** The number of participants included in each site. **Table S2.** The number of participants that completed the neuropsychological tests. **Table S3.** Key imaging parameters in each site. **Table S4.** Group differences of MRI-based measures. **Table S5.** Go biological processes associated with the gene sets explaining variance of the cerebellar functional alterations in MS and NMOSD.

## Data Availability

The transcriptional data are publicly available from the AHBA dataset (http://human.brain-map.org/). Other data are available from the corresponding author on reasonable request.

## Abbreviations

AC1: Association cortex
AC2: Association cortex
AHBA: Allen Human Brain Atlas
AQP4: Aquaporin-4
BVMT: Brief Visuospatial Memory Test
CVLT: California Verbal Learning Test
DAN: Dorsal attention network
DMN: Default mode network
EDSS: Expanded Disability Status Scale
FDR: False discovery rate
FPN: Frontoparietal network
GO: Gene ontology
IC: Insular cortex
LB: Limbic regions
LN: Limbic network
MNI: Montreal Neurological Institute
MS: Multiple sclerosis
MS-related increases: MS > HCs
MS-related decreases: MS < HCs
MS-specific increases: MS > [NMOSD = HCs]
MS-specific decreases: MS < [NMOSD = HCs]
NMOSD: Neuromyelitis optica spectrum disorder
NMOSD-related increases: NMOSD > HCs
NMOSD-related decreases: NMOSD < HCs
NMOSD-specific increases: NMOSD > [MS = HCs]
NMOSD-specific decreases: NMOSD < [MS = HCs]
PASAT: Paced auditory serial addition task
PLS: Partial least-squares
PM: Primary motor cortex
PMA: Cerebellar Primary Motor A module
PMB: Cerebellar Primary Motor B module
PNM: Cerebellar Primary Non-Motor module
PS: Primary sensory cortex
PSS: Primary/secondary sensory
ROIs: Regions of interest
rs-fMRI: Resting-state functional MRI
SM: Cerebellar Secondary Motor module
SMN: Somatomotor network
SNM: Cerebellar Secondary Non-Motor module
SVM: Support vector machines
VAN: Ventral attention network
VN: Visual network
WM: White matter
