# Supplementary material for: Cerebellar connectome alterations and associated genetic signatures in multiple sclerosis and neuromyelitis optica spectrum disorder
Source: J Transl Med. 2023 May 27;21:352. doi: 10.1186/s12967-023-04164-w (PMC10225093; doi:10.1186/s12967-023-04164-w)
Supplement: Supplementary file 1 — Additional file 1: Supplementary Methods. Supplementary Results. Supplementary Discussion. Figure S1. Illustrative representation of cerebellar lobules. Figure S2. The stability of cerebellar modular architecture over different combinations of inter-layer connectivity strength ($\upomega$ω) and module resolution ($\upgamma$γ). Figure S3. Alterations in cerebellar module-based cortical thickness. Figure S4. Relationships between cerebellar module-based cortical thickness and disease duration, lesion volume and EDSS in MS. Table S1. The number of participants included in each site. Table S2. The number of participants that completed the neuropsychological tests. Table S3. Key imaging parameters in each site. Table S4. Group differences of MRI-based measures. Table S5. Go biological processes associated with the gene sets explaining variance of the cerebellar functional alterations in MS and NMOSD. [file 12967_2023_4164_MOESM1_ESM.docx]

**Supplementary Materials**

**Supplementary Methods**

**Structural image preprocessing and cortical thickness estimation**

***Cerebellum*.** The structural image preprocessing and cortical thickness estimation of the cerebellum was accomplished by a patch-based multi-atlas segmentation tool called CERES^1^ - the winner of the Medical Image Computing and Computer-Assisted Intervention cerebellum segmentation challenge^2^ - on the web-based platform volBrain (http://volbrain.upv.es).^3^ Briefly, a spatially adaptive non-local means filter was first applied to reduce noise in the structural images. Then, the N4 bias field correction^4^ was performed to correct intensity inhomogeneity. The corrected images were further linearly registered (affine transform) to the standard Montreal Neurological Institute (MNI) space using the Advanced Normalization Tools, followed by the N4 bias field correction again. Subsequent analyses were limited to the cerebellar areas to reduce the computational burden. To achieve a better cerebellum anatomic matching between the corrected images and the library of manually labeled templates, the non-linear transformations were estimated for the corrected images and templates to the MNI152 atlas. By concatenating the forward non-linear transformation to the MNI152 atlas of each library case and the inverse non-linear transformation of each corrected image, a subject-specific library was obtained. Finally, a local intensity normalization based on regions of interest (ROIs) derived from majority voting segmentation of the subject-specific library was applied to ensure the same intensity of cerebellar tissues across participants. After these steps, a voxel-wise cortical thickness map was derived for each participant in the MNI space.

***Cerebrum***. The structural image preprocessing and cortical thickness estimation of the cerebrum was performed by the CAT12 toolbox (http://dbm.neuro.uni-jena.de/cat12/) based on the SPM12 package (https://www.fil.ion.ucl.ac.uk/spm/software/spm12/). As an efficient and reliable alternative to FreeSurfer, the CAT12 toolbox provides a volume-based approach for cortical thickness estimation without extensive reconstruction of cortical surface. The CAT12 started with an initial segmentation of individual structural images into gray matter, white matter (WM) and cerebrospinal fluid based on an adaptive Maximum A Posterior technique.^5^ Then, a projection-based mothed^6^ was performed to estimate cortical thickness which could handle the partial volume information, sulcal blurring and sulcal asymmetries. Subsequently, a central cortical surface was created and reparametrized into a common coordinate system through spherical mapping.^7^ Finally, individual cortical thickness maps were resampled into the common fsaverage template and smoothed using a Gaussian kernel with 15-mm full width at half maximum.

**Functional image preprocessing**

The rs-fMRI data were preprocessed with the SPM12 package (http://www.fil.ion.ucl.ac.uk/spm/software/spm12/). Firstly, following removal of the first 5 volumes for magnetic saturation, inter-volume head motion was corrected through rigid transformation. After converting rotational displacements from degrees to millimeters on the surface of a sphere of radius 50 mm,^8^ participants with excessive motion were excluded in terms of the criteria of > 3 mm translation or > 0.5 mm mean frame-wise displacement. For the remaining participants, there were no significant differences in the maximum [healthy controls (HCs) = 0.675 (IQR = 0.620), multiple sclerosis (MS) = 0.688 (IQR = 0.600), and neuromyelitis optica spectrum disorder (NMOSD) = 0.641 (IQR = 0.552); *p* = 0.287, permutation test] and mean frame-wise [HCs = 0.150 (IQR = 0.102), MS = 0.137 (IQR = 0.099) and NMOSD = 0.139 (IQR = 0.116); *p* = 0.529, permutation test] displacement of head motion among the three groups. Then, the corrected functional images underwent band-pass filtering (0.01 - 0.08 Hz) and nuisance regression [24-parameter head motion profiles,^9^ WM signals, cerebrospinal fluid signals and global signals] in a single linear model to avoid reintroducing artifacts.^10^ The WM and cerebrospinal fluid signals were calculated within subject-specific masks derived from tissue segmentation of individual structural images (threshold = 0.9), which were co-registered to the corresponding mean volume of the corrected functional images. Finally, the functional images were normalized into standard MNI space by applying deformation fields derived from the tissue segmentation of individual structural images, and spatially smoothed by a Gaussian kernel with 6-mm full width at half maximum.

**Removal of site effects on image-based measurements**

For multisite studies, a crucial step is to remove site effects to avoid spurious findings.^11^ In this study, we utilized a harmonization approach called Combat^12^ to moderate site effects. The Combat harmonization is demonstrated to successfully remove inter-site technical variability while preserving inter-site biological variability in image-based measurements. Specifically, the Combat model can be written as:

$y_{ijv}=\alpha_{v}+X_{ij}\beta_{v}+\gamma_{iv}+\delta_{iv}\varepsilon_{ijv}$ (1)

where $y_{ijv}$ represents connectivity strength (or cortical thickness) of edge (or region) $v$ for subject $j$ in site $i$, $\alpha_{v}$ is the average connectivity strength (or cortical thickness) for edge (or region) $v$, $X$ is a design matrix for the covariates of interest (e.g., age, sex and group label), $\beta_{v}$ is a vector of regression coefficients corresponding to covariates in $X$, and $\varepsilon_{ijv}$ is the residual term that is assumed to follow a normal distribution with zero mean. The terms $\gamma_{iv}$ and $\delta_{iv}$ represent the additive and multiplicative site effects of site $i$ on edge (or region) $v$, respectively, and are estimated by conditional posterior means as described in previous studies.^13,14^ The final ComBat-harmonized connectivity strength (or cortical thickness) for edge (or region) $v$ is calculated as:

$y_{ijv}^{ComBat}=\frac{y_{ijv}-\hat{\alpha}_{v}-X_{ij}\hat{\beta}_{v}-\gamma_{iv}^{*}}{\delta_{iv}^{*}}+\hat{\alpha}_{v}+X_{ij}\hat{\beta}_{v}$ (2)

where $\gamma_{iv}^{*}$and $\delta_{iv}^{*}$ are the empirical Bayes estimates of $\gamma_{iv}$ and $\delta_{iv}$, respectively.

**Cerebellar module detection**

Cerebellar modular architecture was identified by applying a multilayer module detection algorithm to a group-level multiplex network,^15^ which integrated morphological and functional connectivity within the cerebellum for the HCs. Firstly, a group-level mean morphological network and functional network were separately obtained for the HCs. Then, a nonparametric method of locally adaptive network sparsification^16^ was utilized such that only those locally significant connections that could not be explained by random variation were retained in the two networks. Treated as two layers, the two networks were subsequently interconnected by adding edges to link each node in one layer with replica of the node in the other layer, therefore forming a multiplex network. Module detection is to find a specific partition of network nodes that yields the largest modularity, $Q$, which is defined for a multilayer network as^15^:

$Q= \frac{1}{2\mu}\sum_{ijsr} \left[ \left( A_{ijs} - \gamma_{s}\frac{k_{is}k_{js}}{2m_{s}} \right)\delta_{sr}+\delta_{ij}C_{jsr} \right]\delta\left( g_{is},g_{jr} \right)$ (3)

where $i$ and $j$ represent nodes, $s$and $r$ represent layers, $A_{ijs}$ denotes intra-layer connectivity strength between node $i$ and node $j$ in layer $s$, $C_{jsr}$ denotes inter-layer connectivity strength of node $j$ between layer $s$ and layer $r$, $k_{is}= \sum_{j} A_{ijs}$ denotes total intra-layer connectivity strength of node$i$ in layer $s$, $m_{s}$ is total connectivity strength of all edges in layer $s$, $\gamma_{s}$ is module resolution in layer $s$ (the larger $\gamma$, the more small-size modules),$g_{is}$/$g_{jr}$ represents module assignment of node $i$/$j$ in layer $s$/$r$, $2\mu$ is equal to$\sum_{jr} \left( k_{js}+ c_{js} \right)$ with $c_{js}=\sum_{r} C_{jsr}$ indicating total inter-layer connectivity strength of node $j$ from layer $s$ to other layers, and $\delta$ is a binary notation (1 if the same node for $\delta_{ij}$, the same layer for $\delta_{sr}$ and the same module assignment for $\delta\left( g_{is},g_{jr} \right)$, and 0 otherwise). In this study, the module detection was performed using the Louvain algorithm.^17^

During the procedures mentioned above, there were two key parameters that may influence the outcome of module detection: the inter-layer connectivity strength, $\omega$, and the module resolution, $\gamma$. To identify stable cerebellar modular architecture, we performed the module detection across a large range in the 2D parameter space of ($\omega$,$\gamma$): $\omega$ = [0.01 - 1] and $\gamma$ = [0.01 - 3] both with an increment of 0.01. Specifically, at each ($\omega$,$\gamma$) in the 2D parameter space, we performed module detection 1,000 times and obtained a consensus matrix in which the elements indicated the proportions of each pair of nodes that were assigned to the same module over the 1,000 repetitions.^18^ If there existed any pair of nodes that were not consistently assigned to the same module, the module detection was further conducted on the consensus matrix (1,000 times) to derive a new consensus matrix. Such procedures were iterated until a consensus partition was obtained. That is, the final consensus matrix included only 0 and 1. After determining cerebellar partition at each ($\omega$,$\gamma$), we employed the variation of information^19^ to evaluate stability of the cerebellar partition over different ($\omega$,$\gamma$). More specifically, we computed the mean variation of information of cerebellar partition between a given ($\omega_{i},\gamma_{j}$) and its eight contiguous neighbors ($\omega_{i-1,}\gamma_{j-1}, \omega_{i-1}{,\gamma}_{j}, \omega_{i-1}{,\gamma}_{j+1}, \omega_{i}{,\gamma}_{j-1}, \omega_{i}{,\gamma}_{j+1}, \omega_{i+1}{,\gamma}_{j-1}, \omega_{i+1}{,\gamma}_{j} and \omega_{i+1,}\gamma_{j+1}$). The smaller the mean variation of information was, the more stable the cerebellar partition was when subtle ($\omega$,$\gamma$) fluctuations occurred. Finally, we searched the widest continuous ($\omega$,$\gamma$) range in which the mean variation of information was equal to the minimum, and the corresponding cerebellar partition was regarded as canonical cerebellar modular architecture.

**Conversion of cerebellar networks from region-level to module-level**

In this study, we first constructed four networks for each participant at the regional level: one 24 × 24 within-cerebellar morphological network, one 24 × 24 within-cerebellar functional network, one 24 × 400 cerebello-cerebral morphological network and one 24 × 400 cerebello-cerebral functional network. Based on the detected cerebellar modular architecture (5 modules; see Results for details) and prior cerebral modules (cerebral cytoarchitectonic modules and cerebral functional modules: cerebrum^C^ and cerebrum^F^ hereafter), the four region-level networks were transformed into six module-level networks: one 5 × 5 within-cerebellar morphological network, one 5 × 5 within-cerebellar functional network, one 5 × 7 cerebello-cerebral^C^ morphological network, one 5 × 7 cerebello-cerebral^C^ functional network, one 5 × 7 cerebello-cerebral^F^ morphological network and one 5 × 7 cerebello-cerebral^F^ functional network. This was achieved by averaging connectivity weights of all connections linking any pair of cerebellar modules or linking one cerebellar module and one cerebral cytoarchitectonic/functional module. In addition, mean cortical thickness was also calculated within each cerebellar module for each participant.

**Statistical analysis**

Chi-squared tests were used to compare dichotomous variables including sex (male vs female), disease state (acute vs chronic) and WM lesion (presence vs absence). For continuous variables (demographic: age; clinical: disease duration and EDSS; neuropsychological: CVLT, BVMT and PASAT; imaging-based: WM lesion volume, cortical thickness, morphological connectivity and functional connectivity), non-parametric permutation tests were utilized due to their non-normal distributions (Lilliefors test). Age, sex and mean framewise displacement of head motion (if applicable) were treated as covariates in the analyses of neuropsychological variables and imaging-based measures. Briefly, for a given continuous variable, we initially calculated a statistic (T or F) through two-sample t test (for disease duration, EDSS and WM lesion volume), ANOVA (for age) or ANCOVA (for CVLT, BVMT, PASAT, cortical thickness, morphological connectivity and functional connectivity). To obtain an empirical distribution of the statistic, we randomly reshuffled the data and re-computed the statistic (10,000 times). Based on the empirical distribution, a *p*-value was calculated as the proportion of permutations that generated the absolute statistic equal to or greater than the real observation. Notably, the covariates were not reshuffled during the permutation process. To correct for multiple comparisons for imaging-based measures, the false discovery rate (FDR) procedure was used at the level of *q* < 0.05 (cortical thickness: 5 cerebellar modules; morphological/functional connectivity: 15 within-cerebellar and 35 cerebello-cerebral connections). For significant differences among the three groups, post hoc pairwise comparisons were further performed by permutation tests.

**Genetic correlates of cerebellar connectivity alterations in MS and NMOSD**

We utilized gene data from the AHBA to explore genetic correlates of cerebellar morphological and functional connectivity alterations in MS and NMOSD. The AHBA provides transcriptional activity of 20,737 genes from 3,702 spatially distinct tissue samples that are distributed across almost the entire brain. The tissue samples were collected from six healthy adult human donors (left hemisphere for 4 donors and both hemispheres for 2 donors). Before dissection, structural MRI images were acquired for each donor which were further spatially normalized to the MNI space. The AHBA gene data were processed with the abagen toolbox (version 0.1.3; https://github.com/rmarkello/abagen),^20^ which provides standardized workflows according to previously established recommendations.^21^ Firstly, we updated the probe-to-gene annotations using up-to-date information. Then, intensity-based filtering was applied to exclude probes that did not exceed background noise in more than 50% of the samples. Afterwards, a representative probe was selected for each gene that had the most consistent pattern of regional variations across the six donor brains as quantified by a measure called Differential Stability.^22,23^ To assign gene expression samples to the cerebellar and cerebral ROIs, we excluded samples further than 2 mm away from any voxel in the ROIs, and assigned each of the remaining samples to its nearest region according to the minimum distance between the sample and any voxel in a region (of note, the cerebellar ROIs were derived from structural MRI images of the donors with the CERES). Meanwhile, gene expression levels of the remaining samples were normalized for each donor by applying a scaled robust sigmoid normalization for every sample across genes and for every gene across samples in order to assess the relative expression of each gene across regions while controlling for donor-specific differences in gene expression. To obtain regional gene expression profiles, the normalized expressions for samples assigned to the same region were averaged for each donor and aggregated into a region × gene matrix comprising of expression levels of 15,631 genes over all regions. The resultant matrices were further averaged for each hemisphere across donors. After these steps, we obtained group-level gene expression profiles for each brain region, quantifying the mean transcriptional activity of 15,631 genes. The group-level regional gene expression profiles were finally used to calculate gene co-expression (Pearson correlation) between each pair of regions within the cerebellum and for each pair of regions between the cerebellum and cerebrum. Similar to morphological and functional brain networks, the region-level gene co-expression network within the cerebellum was further converted into one 5 × 5 network, and the cerebello-cerebral gene co-expression network was further converted into one 5 × 7 cerebello-cerebral^C^ network and one 5 × 7 cerebello-cerebral^F^ network based on cerebellar modules and/or cerebral partitions.

Partial least-squares (PLS) regression was used to separately examine the relationship of cerebellar connectivity (morphological and functional) alterations in the patients (MS and NMOSD) with genes. In the PLS regression model, the response variable was cerebellar connectivity alterations both within the cerebellum and between the cerebellum and cerebrum (quantified by -log_10_(*p*)*sign(*t*)), and the predictor variables were the contributions of each of the 15,631 genes to gene co-expression of regions in the cerebellum. For a given gene, the contribution was defined as the difference matrix between the gene co-expression network estimated from all genes and gene co-expression network estimated from all genes after excluding the given gene. Elements in the difference matrix therefore indicate the extent to which the given gene induces perturbations in or contributes to gene co-expression of regions within the cerebellum. The first component of the PLS (PLS1) was the linear combination of the contributions of all genes to gene co-expression of regions in the cerebellum that exhibited the strongest correlation with cerebellar connectivity alterations. Significance levels of the correlations were estimated through Moran spectral randomization to account for spatial autocorrelation (10,000 times).^24^ To identify the most relevant genes for cerebellar connectivity alterations, the weight of each gene to form the PLS1 was converted to a Z score by subtracting the mean value and dividing by the standard deviation of all gene weights, and genes with an absolute Z score greater than 1.64 were considered to significantly related to cerebellar connectivity alterations.

To better understand the identified genes, we further performed a gene ontology (GO) functional enrichment analysis to identify the gene-related biological processes. Specifically, for genes that were positively/negatively (i.e. PLS1+/- genes) related to cerebellar connectivity alterations, the GO functional enrichment analysis was conducted using the online tool GOrilla (http://cbl-gorilla.cs.technion.ac.il, version 27 Mar 2022)^25^ with all 15,631 genes as the background list. Significance level of a GO term was estimated based on the number of genes associated with that GO term. Significant GO terms were determined after correcting for multiple comparisons with the FDR procedure at the level of q < 0.05. After removal of general GO terms associated with more than 1,000 genes in the background list,^26,27^ the significant GO terms were summarized by removing redundant GO terms with the online tool REViGO (http://revigo.irb.hr, version 27 Mar 2022).^28^

Finally, we examined the cell-type specificity in the spatial expression distribution of the identified genes in seven canonical cell classes. Specifically, we counted the numbers of the identified genes in each of seven canonical cell classes: excitatory neurons, inhibitory neurons, oligodendrocyte progenitors, astrocytes, endothelial cells, microglia and oligodendrocytes.^29^ To determine whether the identified genes preferentially locate in specific cell classes, we randomly selected *n* genes (*n*, the number of identified genes) and recorded their frequencies in different cell classes. After 10,000 repetitions, a null distribution was obtained for each cell class based on which a p-value was calculated as the proportion of random gene sets for which the resultant numbers of genes in a cell class exceeded or equaled the number of identified genes in the same cell class. The FDR procedure was used to correct for multiple comparisons across cell classes at the level of q < 0.05.

**Classification analysis**

To examine the potential of cerebellar connectivity in distinguishing the three groups from each other, we trained linear SVM classifiers with all within-cerebellar and cerebello-cerebral morphological and functional connectivity as initial features. Out-of-sample classification performance of the classifiers was evaluated using a 10-fold cross-validation procedure. Specifically, in each fold of classification between two groups, feature selection was first performed to exclude irrelevant features through non-parametric permutation tests on the training dataset (*p* < 0.05; 1,000 permutations). Based on the selected features, a linear SVM classifier was then trained and applied to the testing dataset. After the 10-fold cross-validation procedure, a classification accuracy was calculated. To obtain robust estimation of the classification accuracy, the above classification process was repeated 100 times and the resultant mean accuracy across all repeats was reported. Meanwhile, the consensus features that were consistently selected in at least 90% of all folds and repeats (10 × 100 = 1,000) were recorded together with their mean weights. Finally, to further evaluate whether the classifiers performed significantly better than random operations, an empirical null distribution of classification accuracy was obtained by reshuffling the group labels of participants (1,000 times) followed by 10-fold cross-validated classification. A *p*-value was computed as the proportion of values in the empirical null distribution that were greater than the real observation. Notably, effects of age, sex, and mean frame-wise head motion (if applicable) were removed from all features by multiple linear regression before the classification procedure.

**Supplementary Results**

**Cerebellar modular architecture**

In the searched 2D parameter space of ($\omega$,$\gamma$), we identified a widest range ($\omega=0.06 \sim0.50$; $\gamma=1.39 \sim2.00$) wherein cerebellar modular architecture maintained stable when the parameters fluctuated (Supplementary Figure 2). Specifically, for each combination of the parameter pair in the identified range, the cerebellum was consistently subdivided into five spatially contiguous and bilaterally symmetrical modules with regions resembling largely between morphological and functional networks (*Q* = 0.566; Figure 1). The modules had a good correspondence with the well-established cerebellar double-motor/triple-non-motor organization, and therefore were termed Primary Motor A module (PMA, including bilateral Lobule I-II and Lobule III, with additional bilateral Lobule X for morphological networks), Primary Motor B module (PMB, including bilateral Lobule IV, Lobule V and Lobule VI), Primary Non-Motor module (PNM, including bilateral Crus I and Crus II), Secondary Motor module (SM, including bilateral Lobule VIIb, Lobule VIIIa and Lobule VIIIb) and Secondary Non-Motor module (SNM, including bilateral lobule IX, with additional bilateral Lobule X for functional networks).

**Alterations in cerebellar module-based cortical thickness**

Non-parametric permutation tests revealed significant group effects in the mean cortical thickness within the cerebellar PMA, PMB, SM and SNM (*p* < 0.05, FDR corrected). Post hoc comparisons revealed that the group effects were due to common cortical thickening to the two patient groups ([MS = NMOSD] > HCs): SM and SNM), MS-specific cortical thickening (MS > [NMOSD = HCs]: PMA), or NMOSD-specific cortical atrophy (NMOSD < [MS = HCs]: PMB) (Supplementary Figure 3 and Supplementary Table 4). Moreover, significant correlations (*p* < 0.05, FDR corrected) were found for the mean cortical thickness within the cerebellar PMA with disease duration (r = 0.193, *p* = 0.006), lesion volume (r = 0.235, *p* < 0.001) and EDSS (r = 0.207, *p* = 0.003), and for the mean cortical thickness within the cerebellar SNM with lesion volume (r = 0.230, *p* < 0.001) among the MS patients (Supplementary Figure 4).

**Differences in cerebellar cortical thickness between the relapsing and remitting phase**

No significant differences were observed in cerebellar cortical thickness between the relapsing and remitting phase in neither MS nor NMOSD no matter we compared the cerebellar regions showing disease-related alterations (MS: 3; NMOSD: 3) or all cerebellar regions (*p* > 0.05, FDR corrected).

**Supplementary Discussion**

**Cerebellar modular architecture**

Modular organization is one of the main organizational principles of the human brain networks.^30^ For the cerebellum, a hierarchical double-motor/triple-non-motor organization has been well established previously based on cerebellar representations of cerebral intrinsic connectivity networks,^31^ cerebellar task-evoked activity,^32^ cerebello-cerebral functional connectivity profiles^32^ and gradients of within-cerebellar functional connectivity patterns.^33^ In contrast to a single modality used in these previous studies, here we merged both morphological and functional connectome information and reliably identified five modules within the cerebellum with modular compositions largely comparable between morphological and functional cerebellar networks. Moreover, the cerebellar modular architecture well matches with the cerebellar double-motor/triple-non-motor organization, and therefore provide further, in particular new anatomical, evidence for the cerebellar hierarchical organization. Specifically, the PMA and PMB correspond to the first motor representation of the cerebellum and are mainly involved in leg and foot movements, and hand and face movements, respectively^34,35^; The PNM is a combination of the first and contiguous second non-motor representations of the cerebellum and is primarily related to working memory, executive function, language, social processing and emotional processing^32,33^; The SM mirrors the second motor representation of the cerebellum and is mainly engaged in motor activities with demands of attention, working memory and visual process^33,36,37^; The SNM resembles the third non-motor representation of the cerebellum and is predominantly engaged in compounded domains including working memory and emotional processing, and sometimes additional visuo-spatial functions and balance maintaining.^32,37,38^

**Alterations in cerebellar module-based cortical thickness**

Our cortical thickness comparisons mainly revealed thickening in the MS and NMOSD patients. This is in contrast to previous cerebellar studies based on gray matter volume that consistently reported decreases in MS and NMOSD.^39–42^ The discrepancy may be due to that volumetric measures may overlook specific morphological alterations since they reflect a composite of cortical thickness, surface area and folding.^43^ Interpretation of cortical thickening is not straightforward. One speculative interpretation is attributable to delays in normal physiological cleaning process caused by disease effects on pruning redundancy.^44^ Another possible reason, which may be more relevant to this study, is compensatory cortical reorganization to gain or maintain function.^45^ Specifically, we found that both the MS and NMOSD patients exhibited cortical thickening in the SM and SNM modules. Given the functions of these two modules, the common cortical thickening might reflect similar compensatory mechanisms in response to functional declines shared by the two diseases in visual scanning, motor speed, attention, learning and working memory.^46,47^ In addition, we found MS-specific cortical thickening in the leg- and foot-related PMA module, which might be due to adaptive reorganization caused by leg restlessness, a frequent and unique sensory symptom in MS patients.^48,49^ Moreover, the mean cortical thickness within the PMA was positively correlated with EDSS scores and disease durations in the MS patients, suggesting that cortical thickening may be a biomarker to monitor clinical disability of MS. Finally, we found NMOSD-specific cortical thinning in the PMB module. Several previous case studies have reported that NMOSD patients presented choreoathetosis or pseudoathetosis, a series of involuntary and irregular movements of the face, head, hand or limbs.^50–52^ Since cortical thinning typically reflects neuron loss and functional deficits, the thinner PMB is speculated to, at least partly, be responsible for these symptoms in NMOSD patients in view of its involvement in hand and face movements.

# Supplementary References

1. Romero JE, Coupé P, Giraud R, et al. CERES: A new cerebellum lobule segmentation method. *NeuroImage*. 2017;147:916-924. doi:10.1016/j.neuroimage.2016.11.003

2. Carass A, Cuzzocreo JL, Han S, et al. Comparing fully automated state-of-the-art cerebellum parcellation from magnetic resonance images. *NeuroImage*. 2018;183:150-172. doi:10.1016/j.neuroimage.2018.08.003

3. Manjón JV, Coupé P. volBrain: An Online MRI Brain Volumetry System. *Front Neuroinformatics*. 2016;10. doi:10.3389/fninf.2016.00030

4. Tustison NJ, Avants BB, Cook PA, et al. N4ITK: improved N3 bias correction. *IEEE Trans Med Imaging*. 2010;29(6):1310-1320. doi:10.1109/TMI.2010.2046908

5. Rajapakse JC, Giedd JN, Rapoport JL. Statistical approach to segmentation of single-channel cerebral MR images. *IEEE Trans Med Imaging*. 1997;16(2):176-186. doi:10.1109/42.563663

6. Dahnke R, Yotter RA, Gaser C. Cortical thickness and central surface estimation. *Neuroimage*. 2013;65:336-348. doi:10.1016/j.neuroimage.2012.09.050

7. Yotter RA, Thompson PM, Gaser C. Algorithms to Improve the Reparameterization of Spherical Mappings of Brain Surface Meshes. *J Neuroimaging*. 2011;21(2):e134-e147. doi:10.1111/j.1552-6569.2010.00484.x

8. Power JD, Barnes KA, Snyder AZ, Schlaggar BL, Petersen SE. Spurious but systematic correlations in functional connectivity MRI networks arise from subject motion. *NeuroImage*. 2012;59(3):2142-2154. doi:10.1016/j.neuroimage.2011.10.018

9. Friston KJ, Williams S, Howard R, Frackowiak RSJ, Turner R. Movement-Related effects in fMRI time-series: Movement Artifacts in fMRI. *Magn Reson Med*. 1996;35(3):346-355. doi:10.1002/mrm.1910350312

10. Lindquist MA, Geuter S, Wager TD, Caffo BS. Modular preprocessing pipelines can reintroduce artifacts into fMRI data. *Hum Brain Mapp*. 2019;40(8):2358-2376. doi:10.1002/hbm.24528

11. Han X, Jovicich J, Salat D, et al. Reliability of MRI-derived measurements of human cerebral cortical thickness: The effects of field strength, scanner upgrade and manufacturer. *NeuroImage*. 2006;32(1):180-194. doi:10.1016/j.neuroimage.2006.02.051

12. Fortin JP, Cullen N, Sheline YI, et al. Harmonization of cortical thickness measurements across scanners and sites. *Neuroimage*. 2018;167:104-120. doi:10.1016/j.neuroimage.2017.11.024

13. Fortin JP, Parker D, Tunç B, et al. Harmonization of multi-site diffusion tensor imaging data. *NeuroImage*. 2017;161:149-170. doi:10.1016/j.neuroimage.2017.08.047

14. Johnson WE, Li C, Rabinovic A. Adjusting batch effects in microarray expression data using empirical Bayes methods. *Biostatistics*. 2007;8(1):118-127. doi:10.1093/biostatistics/kxj037

15. Mucha PJ, Richardson T, Macon K, Porter MA, Onnela JP. Community Structure in Time-Dependent, Multiscale, and Multiplex Networks. *Science*. 2010;328(5980):876-878. doi:10.1126/science.1184819

16. Foti NJ, Hughes JM, Rockmore DN. Nonparametric Sparsification of Complex Multiscale Networks. Rapallo F, ed. *PLoS ONE*. 2011;6(2):e16431. doi:10.1371/journal.pone.0016431

17. Blondel VD, Guillaume JL, Lambiotte R, Lefebvre E. Fast unfolding of communities in large networks. *J Stat Mech Theory Exp*. 2008;2008(10):P10008. doi:10.1088/1742-5468/2008/10/P10008

18. Bassett DS, Porter MA, Wymbs NF, Grafton ST, Carlson JM, Mucha PJ. Robust detection of dynamic community structure in networks. *Chaos Interdiscip J Nonlinear Sci*. 2013;23(1):013142. doi:10.1063/1.4790830

19. Meilă M. Comparing Clusterings by the Variation of Information. In: Schölkopf B, Warmuth MK, eds. *Learning Theory and Kernel Machines*. Vol 2777. Lecture Notes in Computer Science. Springer Berlin Heidelberg; 2003:173-187. doi:10.1007/978-3-540-45167-9_14

20. Markello RD, Arnatkeviciute A, Poline JB, Fulcher BD, Fornito A, Misic B. Standardizing workflows in imaging transcriptomics with the abagen toolbox. *eLife*. 2021;10:e72129. doi:10.7554/eLife.72129

21. Arnatkevic̆iūtė A, Fulcher BD, Fornito A. A practical guide to linking brain-wide gene expression and neuroimaging data. *NeuroImage*. 2019;189:353-367. doi:10.1016/j.neuroimage.2019.01.011

22. Hawrylycz M, Miller JA, Menon V, et al. Canonical genetic signatures of the adult human brain. *Nat Neurosci*. Published online 2015:15.

23. Kirsch L, Chechik G. On Expression Patterns and Developmental Origin of Human Brain Regions. *PLOS Comput Biol*. Published online 2016:25.

24. Wagner HH, Dray S. Generating spatially constrained null models for irregularly spaced data using M oran spectral randomization methods. O’Hara RB, ed. *Methods Ecol Evol*. 2015;6(10):1169-1178. doi:10.1111/2041-210X.12407

25. Eden E, Navon R, Steinfeld I, Lipson D, Yakhini Z. GOrilla: a tool for discovery and visualization of enriched GO terms in ranked gene lists. *BMC Bioinformatics*. 2009;10(1):48. doi:10.1186/1471-2105-10-48

26. Vértes PE, Rittman T, Whitaker KJ, et al. Gene transcription profiles associated with inter-modular hubs and connection distance in human functional magnetic resonance imaging networks. *Philos Trans R Soc B Biol Sci*. 2016;371(1705):20150362. doi:10.1098/rstb.2015.0362

27. Whitaker KJ, Vértes PE, Romero-Garcia R, et al. Adolescence is associated with genomically patterned consolidation of the hubs of the human brain connectome. *Proc Natl Acad Sci U S A*. 2016;113(32):9105-9110. doi:10.1073/pnas.1601745113

28. Supek F, Bošnjak M, Škunca N, Šmuc T. REVIGO Summarizes and Visualizes Long Lists of Gene Ontology Terms. *PLoS ONE*. 2011;6(7):e21800. doi:10.1371/journal.pone.0021800

29. Arnatkeviciute A, Fulcher BD, Oldham S, et al. Genetic influences on hub connectivity of the human connectome. *Nat Commun*. 2021;12(1):4237. doi:10.1038/s41467-021-24306-2

30. Sporns O, Betzel RF. Modular Brain Networks. *Annu Rev Psychol*. 2016;67(1):613-640. doi:10.1146/annurev-psych-122414-033634

31. Buckner RL, Krienen FM, Castellanos A, Diaz JC, Yeo BTT. The organization of the human cerebellum estimated by intrinsic functional connectivity. *J Neurophysiol*. 2011;106(5):2322-2345. doi:10.1152/jn.00339.2011

32. Guell X, Gabrieli JDE, Schmahmann JD. Triple representation of language, working memory, social and emotion processing in the cerebellum: convergent evidence from task and seed-based resting-state fMRI analyses in a single large cohort. *NeuroImage*. 2018;172:437-449. doi:10.1016/j.neuroimage.2018.01.082

33. Guell X, Schmahmann JD, Gabrieli JD, Ghosh SS. Functional gradients of the cerebellum. *eLife*. 2018;7:e36652. doi:10.7554/eLife.36652

34. Marek S, Siegel JS, Gordon EM, et al. Spatial and Temporal Organization of the Individual Human Cerebellum. *Neuron*. 2018;100(4):977-993.e7. doi:10.1016/j.neuron.2018.10.010

35. Stoodley CJ, Schmahmann JD. Evidence for topographic organization in the cerebellum of motor control versus cognitive and affective processing. *Cortex*. 2010;46(7):831-844. doi:10.1016/j.cortex.2009.11.008

36. Brissenden JA, Tobyne SM, Osher DE, Levin EJ, Halko MA, Somers DC. Topographic Cortico-cerebellar Networks Revealed by Visual Attention and Working Memory. *Curr Biol*. 2018;28(21):3364-3372.e5. doi:10.1016/j.cub.2018.08.059

37. Palesi F, Ferrante M, Gaviraghi M, et al. Motor and higher‐order functions topography of the human dentate nuclei identified with tractography and clustering methods. *Hum Brain Mapp*. 2021;42(13):4348-4361. doi:10.1002/hbm.25551

38. Barmack NH, Pettorossi VE. Adaptive Balance in Posterior Cerebellum. *Front Neurol*. 2021;12:635259. doi:10.3389/fneur.2021.635259

39. Calabrese M, Mattisi I, Rinaldi F, et al. Magnetic resonance evidence of cerebellar cortical pathology in multiple sclerosis. *J Neurol Neurosurg Psychiatry*. 2010;81(4):401-404. doi:10.1136/jnnp.2009.177733

40. Cocozza S, Pontillo G, Russo C, et al. Cerebellum and cognition in progressive MS patients: functional changes beyond atrophy? *J Neurol*. 2018;265(10):2260-2266. doi:10.1007/s00415-018-8985-6

41. Grothe M, Lotze M, Langner S, Dressel A. Impairments in Walking Ability, Dexterity, and Cognitive Function in Multiple Sclerosis Are Associated with Different Regional Cerebellar Gray Matter Loss. *The Cerebellum*. 2017;16(5-6):945-950. doi:10.1007/s12311-017-0871-8

42. Sun J, Zhang N, Wang Q, et al. Normal-Appearing Cerebellar Damage in Neuromyelitis Optica Spectrum Disorder. *Am J Neuroradiol*. 2019;40(7):1156-1161. doi:10.3174/ajnr.A6098

43. Hutton C, Draganski B, Ashburner J, Weiskopf N. A comparison between voxel-based cortical thickness and voxel-based morphometry in normal aging. *NeuroImage*. 2009;48(2):371-380. doi:10.1016/j.neuroimage.2009.06.043

44. Hong SJ, Bernhardt BC, Schrader DS, Bernasconi N, Bernasconi A. Whole-brain MRI phenotyping in dysplasia-related frontal lobe epilepsy. *Neurology*. 2016;86(7):643-650. doi:10.1212/WNL.0000000000002374

45. Burge WK, Griffis JC, Nenert R, et al. Cortical thickness in human V1 associated with central vision loss. *Sci Rep*. 2016;6(1):23268. doi:10.1038/srep23268

46. Guimarães J, Sá MJ. Cognitive Dysfunction in Multiple Sclerosis. *Front Neurol*. 2012;3. doi:10.3389/fneur.2012.00074

47. Oertel. Oertel_2019_FN_Cognitive Impairment in Neuromyelitis Optica Spectrum.pdf. *Front Neurol*. Published online 2019.

48. Manconi M, Fabbrini M, Bonanni E, et al. High prevalence of restless legs syndrome in multiple sclerosis. *Eur J Neurol*. 2007;14(5):534-539. doi:10.1111/j.1468-1331.2007.01740.x

49. Schürks M, Bussfeld P. Multiple sclerosis and restless legs syndrome: a systematic review and meta-analysis. *Eur J Neurol*. 2013;20(4):605-615. doi:10.1111/j.1468-1331.2012.03873.x

50. Boddu A, Shenker J. The Hand that Danced: A Case of Seronegative NMO presenting with Pseudoathetosis (P2.2-048). *Neurology*. 2019;92(15 Supplement):P2.2-048.

51. Seok HY, Jang SH, You S. Neuromyelitis Optica Spectrum Disorder Presenting with Pseudoathetosis. *J Clin Neurol*. 2018;14(1):123. doi:10.3988/jcn.2018.14.1.123

52. Sugeno N, Izumi R, Kato K, Nakashima I, Takeda A, Aoki M. Choreoathetosis in a patient with neuromyelitis optica spectrum disorder. *Neurol Clin Neurosci*. 2013;1(4):154-156. doi:10.1111/ncn3.36


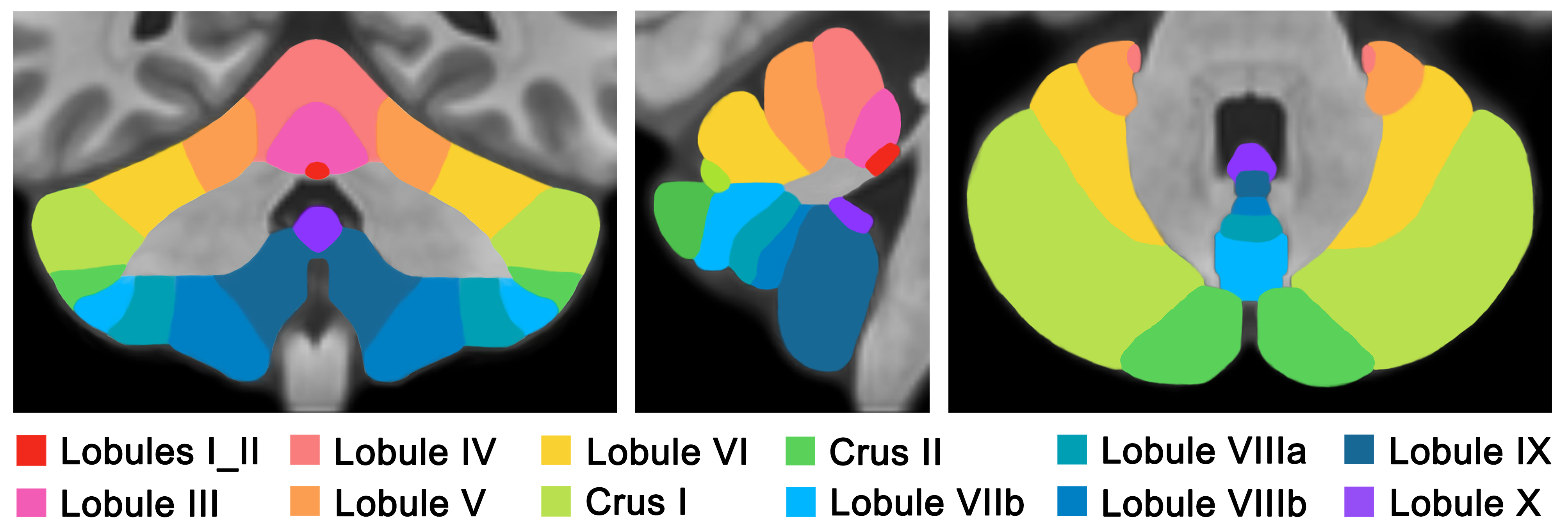


**Supplementary Figure 1.** Illustrative representation of cerebellar lobules. According to the CERES partition, the cerebellum was segmented into 12 lobules in each hemisphere.


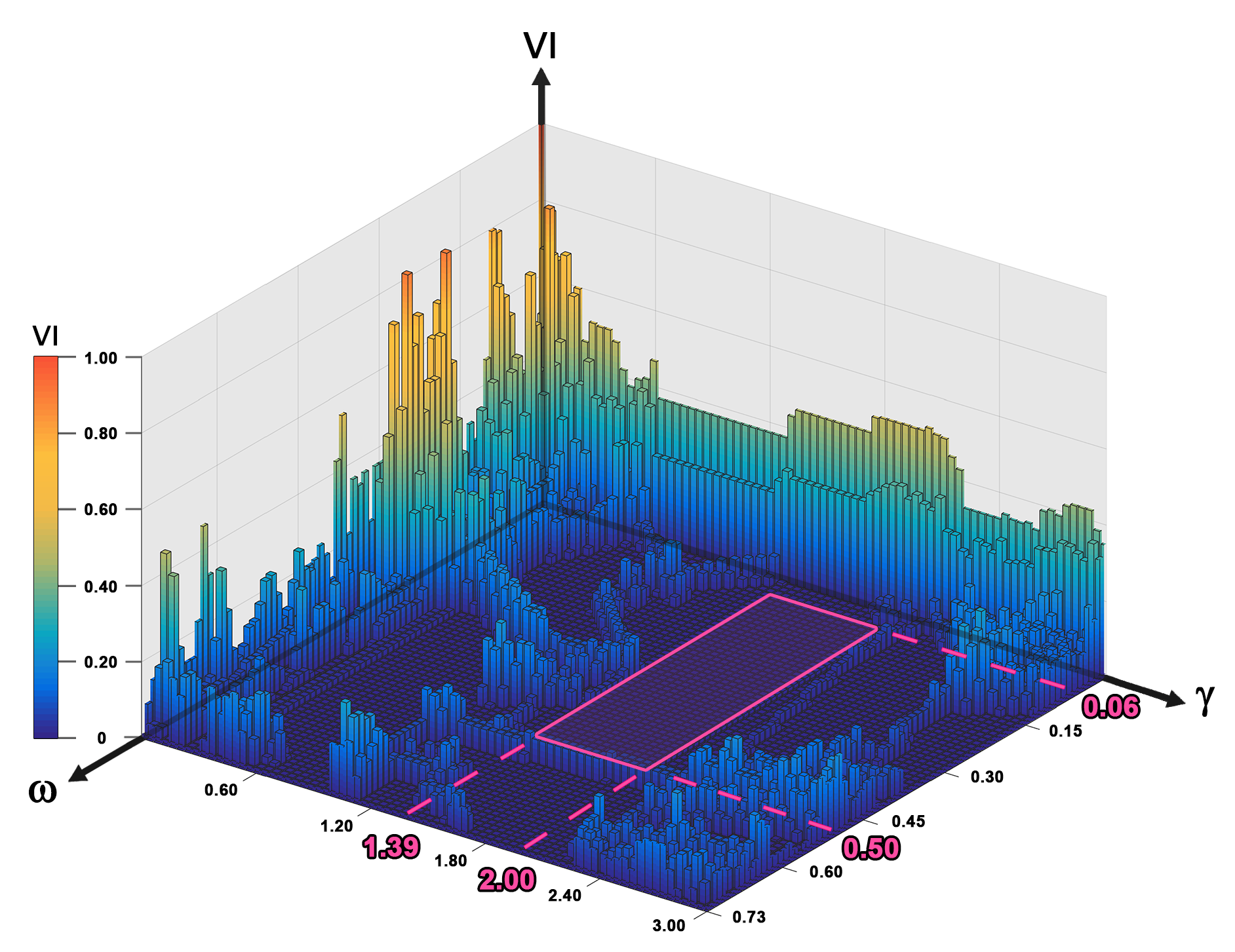


**Supplementary Figure 2.** The stability of cerebellar modular architecture over different combinations of inter-layer connectivity strength ($\omega$) and module resolution ($\gamma$). The variation of information was used to evaluate the stability of cerebellar modular architecture. In the searched 2D parameter space ($\omega$ = [0.01 - 1]; $\gamma$ = [0.01 - 3]), a widest range ($\omega=0.06 \sim0.50$; $\gamma=1.39 \sim2.00$) was identified wherein cerebellar modular architecture maintained stable when the parameters fluctuated. VI, variation of information.


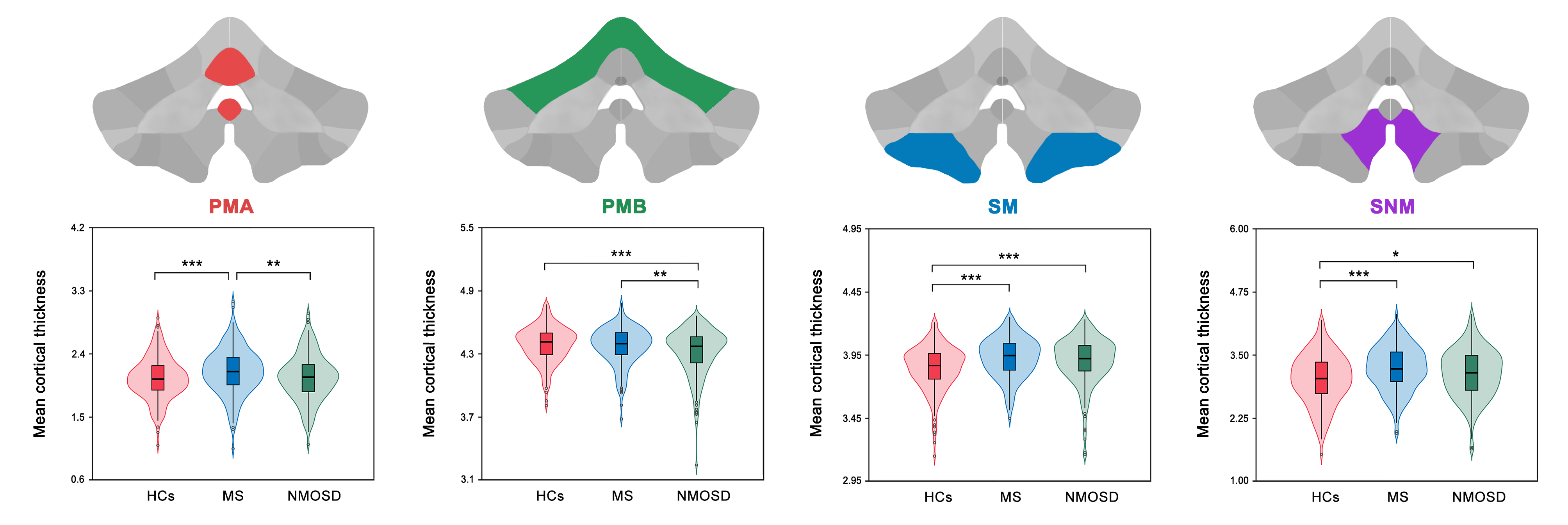


**Supplementary Figure 3.** Alterations in cerebellar module-based cortical thickness. Significant group effects were found on mean cortical thickness within four cerebellar modules (PMA: MS-specific cortical thickening; PMB: NMO-specific cortical atrophy; SM and SNM: common cortical thickening to the two patient groups). HCs, healthy controls; MS, multiple sclerosis; NMOSD, neuromyelitis optica spectrum disorders; PMA, Primary Motor A; PMB, Primary Motor B; SM, Secondary Motor; SNM, Secondary Non-Motor; *, *p* < 0.05; **, *p* < 0.01; ***, *p* < 0.001.


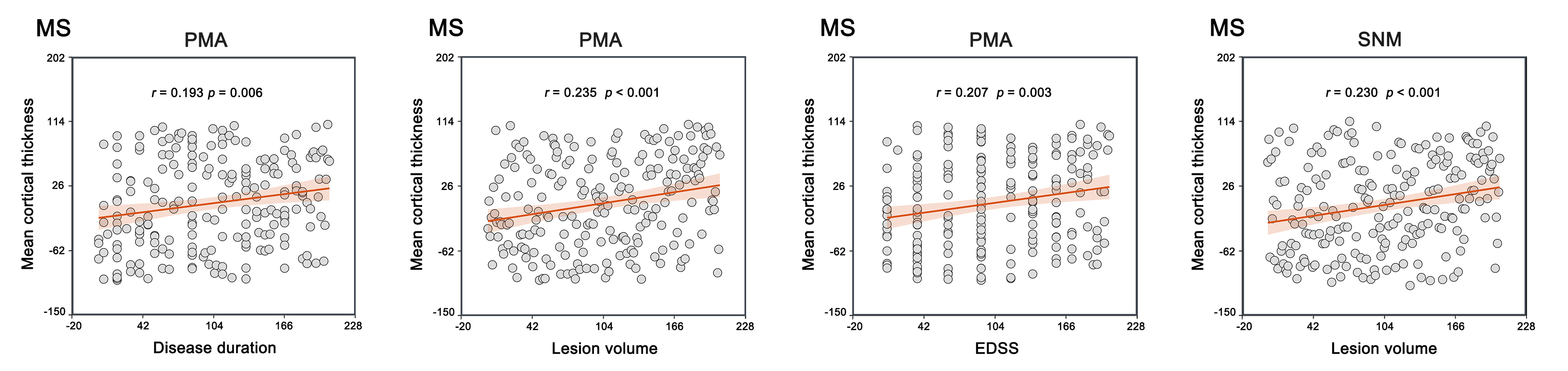


**Supplementary Figure 4.** Relationships between cerebellar module-based cortical thickness and disease duration, lesion volume and EDSS in MS. Among the MS patients, significantly positive correlations were found for mean cortical thickness within the PMA with disease duration, lesion volume and EDSS, and for mean cortical thickness within the SNM with lesion volume. MS, multiple sclerosis; EDSS, Expanded Disability Status Scale; PMA, Primary Motor A; SNM, Secondary Non-Motor; EDSS, expanded disability status scale.

**Supplementary Table 1.** The number of participants included in each site

| Site | HCs  (n = 228) | MS  (n = 208) | NMOSD  (n = 200) |
| --- | --- | --- | --- |
| CQ | 29 | 25 | 20 |
| HS | 33 | 35 | 28 |
| JL | 0 | 15 | 9 |
| NC | 74 | 33 | 5 |
| TJ | 23 | 19 | 7 |
| XW | 61 | 70 | 106 |
| TT | 8 | 11 | 25 |

CQ, The First Affiliated Hospital of Chongqing Medical University, Chongqing; HS, Huashan Hospital Affiliated to Fudan University, Shanghai; JL, China-Japan union hospital of Jilin university, Changchun, Jinlin Province; NC, The first affiliation hospital of Nan Chang university, Nanchang, Jiangxi Province; TJ, Tianjin Medical University General Hospital, Tianjin; XW, Beijing Xuanwu hospital, Capital Medical University, Beijing; TT, Beijing Tiantan hospital, Capital Medical University, Beijing; HCs, healthy controls; MS, multiple sclerosis; NMOSD, neuromyelitis optica spectrum disorders.

**Supplementary Table 2.** The number of participants that completed the neuropsychological tests

| Site/Group | CVLT  (n = 145) | BVMT  (n = 160) | PASAT  (n = 246) |
| --- | --- | --- | --- |
| TJ | 30 | 35 | 45 |
| XW | 83 | 91 | 174 |
| TT | 32 | 34 | 27 |
| HC | 43 | 31 | 58 |
| MS (relapsing/remitting) | 30 (9/14) | 51 (12/30) | 84 (20/54) |
| NMOSD (relapsing/remitting) | 72 (20/49) | 78 (20/54) | 104 (23/77) |

Of note, the information of disease phase (relapsing/remitting) is missing for some patients. CVLT, California Verbal Learning Test; BVMT, Brief Visuospatial Memory Test; PASAT, Paced Auditory Serial Addition Test; TJ, Tianjin Medical University General Hospital, Tianjin; XW, Beijing Xuanwu hospital, Capital Medical University, Beijing; TT, Beijing Tiantan hospital, Capital Medical University, Beijing; HCs, healthy controls; MS, multiple sclerosis; NMOSD, neuromyelitis optica spectrum disorders.

**Supplementary Table 3.** Key imaging parameters in each site

| Site | Scanner | FA  (degree) | | TR/TE  (ms) | Voxel size  (mm^3^) | Matrix size | # of volume |
| --- | --- | --- | --- | --- | --- | --- | --- |
| **Functional image** | | | | | | | |
| CQ | GE Discovery MR750 | | 90 | 2000/40 | 3.75×3.75×4 | 64×64×33 | 240 |
| HS | GE Discovery MR750 | | 90 | 2000/30 | 3.75×3.75×4 | 64×64×35 | 210 |
| JL | Siemens Skyra | | 90 | 2500/30 | 3×3×3 | 70×70×43 | 200 |
| NC | Siemens Skyra | | 90 | 2000/22 | 3.5×3.5×4 | 64×64×33 | 240 |
| TJ | GE Discovery MR750 | | 90 | 2000/45 | 3.5×3.5×4.5 | 64×64×32 | 180 |
| XW | Siemens TrioTim | | 90 | 2000/30 | 3.5×3.5×4 | 64×64×32 | 180 |
| TT | Philips Ingenia CX | | 78 | 2000/30 | 3×3×4 | 80×80×40 | 180 |
| **Structural image** | | | | | | | |
| CQ | GE Discovery MR750 | | 12 | 8.3/3.3 | 0.5×0.5×1 | 512×512×186 | - |
| HS | GE Discovery MR750 | | 12 | 8.2/3.2 | 1×1×1 | 256×256×196 | - |
| JL | Siemens Skyra | | 8 | 2300/2.3 | 1×1×1 | 256×256×192 | - |
| NC | Siemens Skyra | | 9 | 1900/2.3 | 1×1×1 | 256×256×176 | - |
| TJ | GE Discovery MR750 | | 12 | 8.2/3.2 | 1×1×1 | 256×256×188 | - |
| XW | Siemens TrioTim | | 9 | 1600/2.1 | 1×1×1 | 256×224×176 | - |
| TT | Philips Ingenia CX | | 8 | 7/3 | 1×1×1 | 256×256×196 | - |

CQ, The First Affiliated Hospital of Chongqing Medical University, Chongqing; HS, Huashan Hospital Affiliated to Fudan University, Shanghai; JL, China-Japan union hospital of Jilin university, Changchun, Jinlin Province; NC, The first affiliation hospital of Nan Chang university, Nanchang, Jiangxi Province; TJ, Tianjin Medical University General Hospital, Tianjin; XW, Beijing Xuanwu hospital, Capital Medical University, Beijing; TT, Beijing Tiantan hospital, Capital Medical University, Beijing; FA, flip angle; TR, repetition time; TE, echo time.

**Supplementary Table 4.** Group differences of MRI-based measures

|  | HCs  (n = 228) | MS  (n = 208) | NMOSD  (n = 200) | *p*-value |
| --- | --- | --- | --- | --- |
| **Cortical thickness** | | | | |
| PMA | 1.920 (0.348) | 2.022 (0.397) | 1.912 (0.402) | 0.002^a,b^ |
| PMB | 4.170 (0.207) | 4.143 (0.204) | 4.098 (0.248) | <0.001^a,c^ |
| SM | 3.963 (0.258) | 4.013 (0.254) | 3.997 (0.242) | <0.001^b,c^ |
| SNM | 3.103 (0.604) | 3.261 (0.540) | 3.181 (0.607) | <0.001^b,c^ |
| **Morphological connectivity** | | | | |
| Within-cerebellar | | | | |
| PMB - PMB | 0.699 (0.025) | 0.692 (0.027) | 0.695 (0.025) | 0.008^b^ |
| SM - SM | 0.678 (0.046) | 0.689 (0.045) | 0.681 (0.041) | 0.006^a,b^ |
| PMA - PMB | 0.463 (0.048) | 0.475 (0.041) | 0.478 (0.049) | 0.003^b,c^ |
| PMA - SM | 0.494 (0.060) | 0.490 (0.063) | 0.477 (0.074) | 0.004^c^ |
| PMA - SNM | 0.683 (0.110) | 0.671 (0.099) | 0.671 (0.108) | <0.001^b,c^ |
| PMB - SNM | 0.659 (0.133) | 0.691 (0.127) | 0.686 (0.136) | <0.001^b,c^ |
| Cerebello-cerebral^C^ | | | | |
| PMB - PM | 0.506 (0.046) | 0.512 (0.048) | 0.518 (0.063) | 0.007^a,c^ |
| PMB - PS | 0.472 (0.042) | 0.476 (0.039) | 0.483 (0.055) | 0.016^a,c^ |
| PMB - LB | 0.504 (0.042) | 0.507 (0.046) | 0.516 (0.052) | 0.004^c^ |
| PMB - IC | 0.497 (0.048) | 0.502 (0.047) | 0.511 (0.057) | <0.001^a,c^ |
| PNM - AC2 | 0.609 (0.068) | 0.600 (0.088) | 0.610 (0.077) | 0.025^b^ |
| SM - PM | 0.527 (0.064) | 0.519 (0.056) | 0.519 (0.046) | 0.002^b,c^ |
| SM - AC1 | 0.533 (0.060) | 0.519 (0.056) | 0.518 (0.050) | <0.001^b,c^ |
| SM - AC2 | 0.526 (0.062) | 0.512 (0.061) | 0.506 (0.054) | <0.001^b,c^ |
| SM - PSS | 0.514 (0.063) | 0.500 (0.057) | 0.497 (0.055) | <0.001^b,c^ |
| SM - PS | 0.496 (0.061) | 0.487 (0.055) | 0.486 (0.049) | <0.001^b,c^ |
| SM - LB | 0.520 (0.057) | 0.514 (0.063) | 0.513 (0.052) | 0.005^b,c^ |
| SM - IC | 0.520 (0.061) | 0.512 (0.064) | 0.512 (0.054) | 0.007^b,c^ |
| SNM - PM | 0.678 (0.075) | 0.665 (0.093) | 0.667 (0.090) | 0.008^b^ |
| SNM - AC1 | 0.680 (0.070) | 0.666 (0.087) | 0.669 (0.083) | 0.002^b,c^ |
| SNM - AC2 | 0.681 (0.079) | 0.662 (0.094) | 0.663 (0.088) | <0.001^b,c^ |
| SNM - PSS | 0.678 (0.078) | 0.655 (0.094) | 0.659 (0.091) | <0.001^b,c^ |
| SNM - PS | 0.662 (0.088) | 0.639 (0.097) | 0.639 (0.093) | 0.002^b,c^ |
| SNM - LB | 0.680 (0.078) | 0.663 (0.088) | 0.666 (0.081) | 0.004^b,c^ |
| SNM - IC | 0.674 (0.076) | 0.658 (0.088) | 0.663 (0.079) | 0.007^b^ |
| Cerebello-cerebral^F^ | | | | |
| PMB - SMN | 0.493 (0.045) | 0.497 (0.044) | 0.504 (0.059) | 0..021^c^ |
| PNM - DMN | 0.623 (0.063) | 0.615 (0.081) | 0.622 (0.069) | 0.022^b^ |
| SM - VN | 0.512 (0.053) | 0.499 (0.059) | 0.498 (0.049) | <0.001^b,c^ |
| SM - SMN | 0.515 (0.059) | 0.505 (0.057) | 0.504 (0.046) | <0.001^b,c^ |
| SM - DAN | 0.515 (0.062) | 0.506 (0.058) | 0.501 (0.054) | <0.001^b,c^ |
| SM - VAN | 0.531 (0.064) | 0.518 (0.057) | 0.513 (0.053) | <0.001^b,c^ |
| SM - LN | 0.528 (0.062) | 0.515 (0.056) | 0.514 (0.059) | <0.001^b,c^ |
| SM - FPN | 0.527 (0.062) | 0.512 (0.056) | 0.509 (0.053) | <0.001^b,c^ |
| SM - DMN | 0.539 (0.058) | 0.523 (0.057) | 0.521 (0.046) | <0.001^b,c^ |
| SNM - VN | 0.671 (0.077) | 0.652 (0.093) | 0.655 (0.088) | <0.001^b,c^ |
| SNM - SMN | 0.672 (0.082) | 0.660 (0.093) | 0.658 (0.089) | 0.006^b,c^ |
| SNM - DAN | 0.672 (0.082) | 0.658 (0.093) | 0.656 (0.090) | 0.002^b,c^ |
| SNM - VAN | 0.679 (0.072) | 0.664 (0.091) | 0.669 (0.083) | 0.002^b,c^ |
| SNM - LN | 0.686 (0.071) | 0.657 (0.089) | 0.662 (0.085) | <0.001^b,c^ |
| SNM - FPN | 0.686 (0.078) | 0.666 (0.095) | 0.667 (0.085) | <0.001^b,c^ |
| SNM - DMN | 0.683 (0.071) | 0.668 (0.083) | 0.668 (0.082) | 0.001^b,c^ |
| **Functional connectivity** | | | | |
| Within-cerebellar | | | | |
| SM - SM | 0.397 (0.116) | 0.371 (0.119) | 0.414 (0.122) | <0.001^a,b^ |
| Cerebello-cerebral^C^ | | | | |
| PMA - AC1 | -0.004 (0.077) | -0.009 (0.066) | -0.023 (0.080) | 0.003^c^ |
| PMB - AC1 | -0.008 (0.071) | -0.021 (0.055) | -0.023 (0.066) | <0.001^b,c^ |
| PMB - AC2 | -0.032 (0.087) | -0.049 (0.078) | -0.050 (0.089) | 0.003^b,c^ |
| SM - AC1 | 0.001 (0.060) | -0.014 (0.063) | -0.014 (0.056) | 0.002^b,c^ |
| Cerebello-cerebral^F^ | | | | |
| PMA - FPN | 0.002 (0.124) | -0.005 (0.119) | -0.033 (0.130) | 0.001^a,c^ |
| PMB - LN | -0.018 (0.104) | -0.008 (0.105) | -0.044 (0.121) | 0.001^a,c^ |
| PMB - FPN | -0.040 (0.139) | -0.068 (0.114) | -0.069 (0.131) | <0.001^b,c^ |
| SM - FPN | 0.014 (0.122) | -0.009 (0.118) | -0.016 (0.098) | <0.001^b,c^ |
| SM - DMN | -0.088 (0.110) | -0.083 (0.101) | -0.113 (0.132) | <0.001^a,c^ |

Data are represented as median (interquartile range). HCs, healthy controls; MS, multiple sclerosis; NMOSD, neuromyelitis optica spectrum disorders; PMA, Primary Motor A; PMB, Primary Motor B; PNM, Primary Non-Motor; SM, Secondary Motor; SNM, Secondary Non-Motor; PM, primary motor cortex; AC1, association cortex; AC2, association cortex; PSS, primary/secondary sensory; PS, primary sensory cortex; LB, limbic regions; IC, insular cortex; VN, visual network; SMN, somatomotor network; DAN, dorsal attention network; VAN, ventral attention network; LN, limbic network; FPN, frontoparietal network; DMN, default mode network.

^a^Significant differences between the two patient groups.

^b^Significant differences between the MS patients and HCs.

^c^Significant differences between the NMOSD patients and HCs.

**Supplementary Table 5.** Go biological processes associated with the gene sets explaining variance of the cerebellar functional alterations in MS and NMOSD

| **GO Term** | **Description** | ***p*-value** | **Enrichment^a^** | **N** | **B** | **n** | **b** |
| --- | --- | --- | --- | --- | --- | --- | --- |
| **MS (within-cerebellar & cerebello-cerebral^F^)** | | | | | | | |
| GO:0031646 | positive regulation of neurological system process | <0.001 | 7.106 | 13967 | 54 | 364 | 10 |
| GO:0045165 | cell fate commitment | <0.001 | 5.090 | 13967 | 98 | 364 | 13 |
| GO:0007218 | neuropeptide signaling pathway | <0.001 | 4.919 | 13967 | 78 | 364 | 10 |
| GO:0007188 | adenylate cyclase-modulating G protein-coupled receptor signaling pathway | <0.001 | 3.942 | 13967 | 146 | 364 | 15 |
| GO:0007187 | G protein-coupled receptor signaling pathway, coupled to cyclic nucleotide second messenger | <0.001 | 3.553 | 13967 | 162 | 364 | 15 |
| GO:0007268 | chemical synaptic transmission | <0.001 | 3.236 | 13967 | 249 | 364 | 21 |
| GO:0044057 | regulation of system process | <0.001 | 2.814 | 13967 | 450 | 364 | 33 |
| GO:0007267 | cell-cell signaling | <0.001 | 2.805 | 13967 | 424 | 364 | 31 |
| GO:0023052 | signaling | <0.001 | 2.730 | 13967 | 492 | 364 | 35 |
| GO:0034765 | regulation of ion transmembrane transport | <0.001 | 2.675 | 13967 | 373 | 364 | 26 |
| GO:0043269 | regulation of ion transport | <0.001 | 2.380 | 13967 | 532 | 364 | 33 |
| GO:0034762 | regulation of transmembrane transport | <0.001 | 2.349 | 13967 | 441 | 364 | 27 |
| GO:0007154 | cell communication | <0.001 | 2.045 | 13967 | 638 | 364 | 34 |
| **NMOSD (within-cerebellar & cerebello-cerebral^C^)** | | | | | | | |
| GO:0014060 | regulation of epinephrine secretion | <0.001 | 23.327 | 13967 | 5 | 479 | 4 |
| GO:0031652 | positive regulation of heat generation | <0.001 | 12.959 | 13967 | 9 | 479 | 4 |
| GO:0098664 | G protein-coupled serotonin receptor signaling pathway | <0.001 | 11.663 | 13967 | 20 | 479 | 8 |
| GO:0033081 | regulation of T cell differentiation in thymus | <0.001 | 9.720 | 13967 | 15 | 479 | 5 |
| GO:0001659 | temperature homeostasis | <0.001 | 7.952 | 13967 | 22 | 479 | 6 |
| GO:0007218 | neuropeptide signaling pathway | <0.001 | 5.981 | 13967 | 78 | 479 | 16 |
| GO:1905954 | positive regulation of lipid localization | <0.001 | 4.860 | 13967 | 72 | 479 | 12 |
| GO:0007565 | female pregnancy | <0.001 | 4.860 | 13967 | 54 | 479 | 9 |
| GO:0044706 | multi-multicellular organism process | <0.001 | 4.556 | 13967 | 64 | 479 | 10 |
| GO:0044703 | multi-organism reproductive process | <0.001 | 4.166 | 13967 | 70 | 479 | 10 |
| GO:0007187 | G protein-coupled receptor signaling pathway, coupled to cyclic nucleotide second messenger | <0.001 | 3.960 | 13967 | 162 | 479 | 22 |
| GO:0007188 | adenylate cyclase-modulating G protein-coupled receptor signaling pathway | <0.001 | 3.595 | 13967 | 146 | 479 | 18 |
| GO:1905952 | regulation of lipid localization | <0.001 | 3.581 | 13967 | 114 | 479 | 14 |
| GO:0007268 | chemical synaptic transmission | <0.001 | 3.513 | 13967 | 249 | 479 | 30 |
| GO:1903522 | regulation of blood circulation | <0.001 | 3.510 | 13967 | 216 | 479 | 26 |
| GO:0050806 | positive regulation of synaptic transmission | <0.001 | 3.140 | 13967 | 130 | 479 | 14 |
| GO:0007267 | cell-cell signaling | <0.001 | 3.026 | 13967 | 424 | 479 | 44 |
| GO:0044057 | regulation of system process | <0.001 | 2.981 | 13967 | 450 | 479 | 46 |
| GO:0023052 | signaling | <0.001 | 2.904 | 13967 | 492 | 479 | 49 |
| GO:0030182 | neuron differentiation | <0.001 | 2.705 | 13967 | 194 | 479 | 18 |
| GO:0007186 | G protein-coupled receptor signaling pathway | <0.001 | 2.483 | 13967 | 646 | 479 | 55 |
| GO:0034765 | regulation of ion transmembrane transport | <0.001 | 2.345 | 13967 | 373 | 479 | 30 |
| GO:0007154 | cell communication | <0.001 | 2.331 | 13967 | 638 | 479 | 51 |
| GO:0010817 | regulation of hormone levels | <0.001 | 2.318 | 13967 | 390 | 479 | 31 |
| GO:0043269 | regulation of ion transport | <0.001 | 2.247 | 13967 | 532 | 479 | 41 |
| GO:0007610 | behavior | <0.001 | 2.117 | 13967 | 427 | 479 | 31 |
| GO:0034762 | regulation of transmembrane transport | <0.001 | 2.116 | 13967 | 441 | 479 | 32 |
| GO:0050877 | nervous system process | <0.001 | 1.914 | 13967 | 594 | 479 | 39 |
| GO:0003008 | system process | <0.001 | 1.859 | 13967 | 957 | 479 | 61 |
| GO:0048870 | cell motility | <0.001 | 1.833 | 13967 | 716 | 479 | 45 |
| GO:0040011 | locomotion | <0.001 | 1.783 | 13967 | 785 | 479 | 48 |
| **NMOSD (within-cerebellar & cerebello-cerebral^F^)** | | | | | | | |
| GO:0021855 | hypothalamus cell migration | <0.001 | 23.807 | 13967 | 4 | 440 | 3 |
| GO:0031652 | positive regulation of heat generation | <0.001 | 14.108 | 13967 | 9 | 440 | 4 |
| GO:0033081 | regulation of T cell differentiation in thymus | <0.001 | 10.581 | 13967 | 15 | 440 | 5 |
| GO:0098664 | G protein-coupled serotonin receptor signaling pathway | <0.001 | 9.523 | 13967 | 20 | 440 | 6 |
| GO:0001662 | behavioral fear response | <0.001 | 8.281 | 13967 | 23 | 440 | 6 |
| GO:0002209 | behavioral defense response | <0.001 | 7.936 | 13967 | 24 | 440 | 6 |
| GO:0033555 | multicellular organismal response to stress | <0.001 | 6.349 | 13967 | 40 | 440 | 8 |
| GO:0071560 | cellular response to transforming growth factor beta stimulus | <0.001 | 5.771 | 13967 | 44 | 440 | 8 |
| GO:0007218 | neuropeptide signaling pathway | <0.001 | 5.697 | 13967 | 78 | 440 | 14 |
| GO:0007565 | female pregnancy | <0.001 | 5.291 | 13967 | 54 | 440 | 9 |
| GO:0007200 | phospholipase C-activating G protein-coupled receptor signaling pathway | <0.001 | 5.079 | 13967 | 50 | 440 | 8 |
| GO:0044706 | multi-multicellular organism process | <0.001 | 4.960 | 13967 | 64 | 440 | 10 |
| GO:1900449 | regulation of glutamate receptor signaling pathway | <0.001 | 4.683 | 13967 | 61 | 440 | 9 |
| GO:0008306 | associative learning | <0.001 | 4.608 | 13967 | 62 | 440 | 9 |
| GO:0044703 | multi-organism reproductive process | <0.001 | 4.535 | 13967 | 70 | 440 | 10 |
| GO:0007187 | G protein-coupled receptor signaling pathway, coupled to cyclic nucleotide second messenger | <0.001 | 4.507 | 13967 | 162 | 440 | 23 |
| GO:0051952 | regulation of amine transport | <0.001 | 4.290 | 13967 | 74 | 440 | 10 |
| GO:0007156 | homophilic cell adhesion via plasma membrane adhesion molecules | <0.001 | 4.177 | 13967 | 114 | 440 | 15 |
| GO:0007189 | adenylate cyclase-activating G protein-coupled receptor signaling pathway | <0.001 | 4.177 | 13967 | 76 | 440 | 10 |
| GO:0007188 | adenylate cyclase-modulating G protein-coupled receptor signaling pathway | <0.001 | 3.914 | 13967 | 146 | 440 | 18 |
| GO:0048871 | multicellular organismal homeostasis | <0.001 | 3.734 | 13967 | 102 | 440 | 12 |
| GO:0071805 | potassium ion transmembrane transport | <0.001 | 3.527 | 13967 | 126 | 440 | 14 |
| GO:0006813 | potassium ion transport | <0.001 | 3.392 | 13967 | 131 | 440 | 14 |
| GO:0007268 | chemical synaptic transmission | <0.001 | 3.315 | 13967 | 249 | 440 | 26 |
| GO:0030182 | neuron differentiation | <0.001 | 2.945 | 13967 | 194 | 440 | 18 |
| GO:1903522 | regulation of blood circulation | <0.001 | 2.939 | 13967 | 216 | 440 | 20 |
| GO:0007267 | cell-cell signaling | <0.001 | 2.920 | 13967 | 424 | 440 | 39 |
| GO:0023052 | signaling | <0.001 | 2.903 | 13967 | 492 | 440 | 45 |
| GO:0044057 | regulation of system process | <0.001 | 2.822 | 13967 | 450 | 440 | 40 |
| GO:0007186 | G protein-coupled receptor signaling pathway | <0.001 | 2.604 | 13967 | 646 | 440 | 53 |
| GO:0043269 | regulation of ion transport | <0.001 | 2.566 | 13967 | 532 | 440 | 43 |
| GO:0007154 | cell communication | <0.001 | 2.388 | 13967 | 638 | 440 | 48 |
| GO:0034765 | regulation of ion transmembrane transport | <0.001 | 2.298 | 13967 | 373 | 440 | 27 |
| GO:0007610 | behavior | <0.001 | 2.230 | 13967 | 427 | 440 | 30 |
| GO:0050877 | nervous system process | <0.001 | 2.084 | 13967 | 594 | 440 | 39 |
| GO:0003008 | system process | <0.001 | 1.791 | 13967 | 957 | 440 | 54 |

GO, gene ontology; PLS, partial least-squares regression. ^a^Enrichment = (b/n)/(B/N), with n denoting the number of genes in the PLS1+ gene set, b denoting the number of genes in the PLS1+ gene set that associated with a specific GO term, N denoting the total number of genes, and B denoting the total number of genes associated with a specific GO term.
